# Functional analysis of the ABCs of eye color in *Helicoverpa armigera* with CRISPR/Cas9-induced mutations

**DOI:** 10.1038/srep40025

**Published:** 2017-01-05

**Authors:** Sher Afzal Khan, Michael Reichelt, David G. Heckel

**Affiliations:** 1 Department of Entomology, Max Planck Institute for Chemical Ecology, Hans-Knöll-Straße 8, Jena, Germany; 2 Department of Biochemistry, Max Planck Institute for Chemical Ecology, Hans-Knöll-Straße 8, Jena, Germany

## Abstract

Many insect pigments are localized in subcellular pigment granules, and transport of pigment precursors from the cytoplasm is accomplished by ABC proteins. *Drosophila melanogaster* has three half-transporter genes (*white, scarlet*, and *brown*, all affecting eye pigments) and *Bombyx mori* has a fourth (*ok*). The White, Brown, Scarlet and Ok proteins each have one transmembrane and one cytoplasmic domain and they heterodimerize to form functional transporters with different substrate specificities. We used CRISPR/Cas9 to create somatic and germ-line knockout mutations of these four genes in the noctuid moth *Helicoverpa armigera*. Somatic knockouts of *white* block pigmentation of the egg, first instar larva and adult eye, but germ-line knockouts of *white* are recessive lethal in the embryo. Knockouts of *scarlet* are viable and produce pigmentless first instar larvae and yellow adult eyes lacking xanthommatin. Knockouts of *brown* show no phenotypic effects on viability or pigmentation. Knockouts of *ok* are viable and produce translucent larval cuticle and black eyes. CRISPR/Cas9-induced mutations are a useful tool for analyzing how essential and non-essential genes interact to produce the diversity of insect pigmentation patterns found in nature.

Two classes of pigments, tryptophan-derived ommochromes and guanine-derived pteridines, contribute to determine the color of insect eyes. Mosquitoes, bugs, flour beetles and bees use only ommochromes^1^,^2^,^3^,^4^, while flies and grasshoppers use both^5^. These pigments are found in pigment granules of specific cells in insects, and mutations affecting eye color can occur in genes encoding biosynthetic enzymes, transporter proteins, or proteins involved in vesicular trafficking and granule formation^6^. Analysis of eye color mutations in *Ephestia kuehniella* provided the first evidence for the “one gene–one enzyme” theory in classical genetics^7^, and ease in visual screening has led to their use as markers for germ-line transformation of species such as *Drosophila melanogaster, Bombyx mori, Aedes aegypti* and *Tribolium castaneum*^3^,^8^,^9^,^10^.

Ommochrome pigments correspond to different colors depending on insect type^11^,^12^. The tryptophan precursor in the ommochrome pathway is converted to 3-hydroxykynurenine which is subsequently incorporated into pigment granules, where ommatins and ommines are hypothesized to be synthesized^13^. Guanine and other purines are imported into pigment granules where they are converted to pteridines^14^. In *D. melanogaster*, ommochromes are brown and pteridines red and yellow^15^, and both contribute to the deep red color of the adult eye. Eyes with only ommatins in the *brown* mutant appear brown, eyes with only pteridines in the *scarlet* mutant appear bright red, and eyes lacking both pigments in the *white* mutant appear white^16^.

The *white, brown*, and *scarlet* genes encode proteins which transport ommochrome and pteridine pathway precursors into pigment granules in the eye. These proteins belong to the ATP binding cassette (ABC) transporter superfamily^17^. ABC transporters move substrates across membranes using energy obtained by ATP hydrolysis. Full transporters are composed of two membrane spanning domains and two cytoplasmic domains which harbor the ATP binding motifs A and B^18^. The White, Brown, and Scarlet proteins each have one membrane-spanning and one cytoplasmic domain^15^,^19^,^20^, and they heterodimerize to form functional transporters with different substrate specificities. In *Drosophila* and *Bombyx,* White and Scarlet dimerize to form the transporter for ommochrome precursors^17^,^21^,^22^; and in *Drosophila*, White and Brown dimerize to form the transporter for pteridine precursors^14^,^17^. *Bombyx* also possesses a fourth gene *ok*, a paralog of *brown*. The Ok protein forms a heterodimer with White to transport uric acid into pigment granules in the larval epidermis^23^. The *ok* mutant “kinshiryu translucent” has an translucent, oily-appearing epidermis due to the absence of urate from the normally white larval skin^23^.

*Helicoverpa armigera* is a generalist noctuid moth, and one of the most injurious pests of agriculture worldwide^24^. Larvae consume plants in 68 different plant families^25^, adults are highly migratory^26^, and the species has a wide distribution in Africa, Asia, Australia, and Europe. *H. armigera* was first reported in Brazil in 2013^27^ and is currently expanding its range in South and North America^6^. This pest has evolved resistance to chemical insecticides throughout its range^28^,^29^,^30^, and recently to transgenic cotton expressing toxins from *Bacillus thuringiensis* in China^31^ and Pakistan^32^.

For investigating gene function in this non-model lepidopteran species (genome size ~340 MB, n-31 chromosomes), RNA interference is of limited utility^33^ due to highly active RNases that degrade double-stranded RNA. Germ-line transformation would be a useful technique to test hypotheses about the function of resistance-causing genes. In the course of developing transformation techniques for this insect, we have investigated eye color mutations as visible markers for detection of successful transformation events. Due to the rarity of spontaneously-occuring mutants in this species and the difficulty of developing inbred strains, we used the CRISPR/Cas9 technique to create mutations in the *white, scarlet, brown*, and *ok* genes, to investigate their phenotypic effects and to evaluate their suitability as visible markers. We have found that some of these mutants in *H. armigera* have phenotypes similar to other species, but in other cases the new mutations are different, either being lethal or having no visible effects. These species-specific effects illustrate the benefits of applying the CRISPR/Cas9 technique directly to the species of interest, rather than inferring mutant properties from other model systems.

## Results

### Induced mutations in the *white* gene in *H. armigera*

To demonstrate the potential of genome editing systems in *H. armigera*, we selected *white* as a candidate gene, because mutant homozygotes of several other insect species are viable and have white eyes^1^,^17^,^34^,^35^. We used the CRISPR/Cas9 system^36^,^37^, combining artificially synthesized mRNA for the Cas9 protein and the cRNA of the guide RNA directed against exon 3 of the *H. armigera white* gene (*Ha-w*, GenBank Accession KU754476, Supplementary Figure S1A and Supplementary Table 1). This mixture was injected into eggs within 2 hours of oviposition, and G0 embryos were visually examined 24 hours post injection.

**Figure 1:**
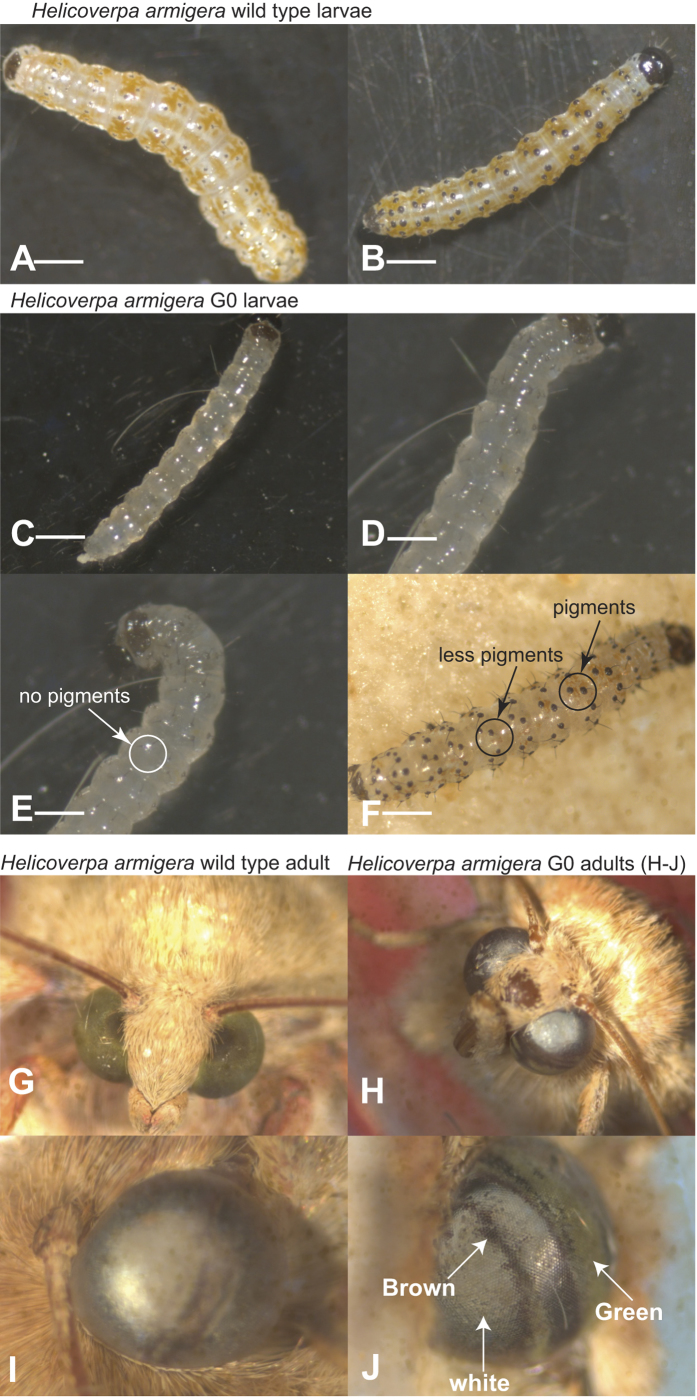
CRISPR/Cas9 induced mutations at the *white* locus in *H. armigera* G0 individuals. (**A,B**) Control larvae have wild type phenotypes at 48 hours post hatching. (**C–F**) The chimeric *Ha-w* mutant larvae at 48 hours post hatching. (**G**) Wild type (WT) eye color in control *H. armigera*. (**H–J**) *Ha-w* mutant adults have mosaic eye color pattern. Scale bar 1mm.

Fertile eggs of this species normally develop a light brown pigmented serosa which forms an irregular equatorial ring covering most of the upper hemisphere at 24 hours (Supplementary Figure S2C). Injected eggs often displayed a broken ring, or remained entirely white. White eggs did not hatch, but some partially pigmented eggs hatched to produce larvae with a mosaic pattern of pigment in the epidermis. The normal first instar larva has a transparent epidermis immediately after hatching, through which the brown-pigmented Malpighian tubules can be seen. After 48 hours, longitudinal stripes appear in the epidermis alternating white (containing urate) and light brown (with ommochromes), and persist throughout the first larval instar (Fig. 1A,B). Some G0 larvae hatching from injected eggs remained entirely clear (Fig. 1C,D), or showed clear patches interrupting this striped pattern (Fig. 1E,F). Entirely clear larvae died at the first or second larval molt, but some mosaic larvae (Supplementary Figure S3) developed to adults. The wild-type adult eye is green with a dark pseudopupil (Fig. 1G). Some G0 adults exhibited mosaic eyes, with stripes of green, brown, or clear ommatidia (Fig. 1H–J). No other changes in larval, pupal or adult scale pigmentation were evident. Some G0 adults could be mated and produced fertile eggs.

DNA was isolated from mosaic G0 individuals or their offspring and PCR amplicons of exon 3 of *white* were cloned and sequenced, revealing numerous small deletions in the targeted region, characteristic of the error-prone non-homologous end-joining repair of the double-stranded DNA breaks caused by the Cas9 nuclease (Supplementary Figure S4). Many crosses of G0 adults produced light brown eggs and wild-type appearing progeny with no evident pigmentation differences, indicating that these induced mutations did not occur in the germ-line. However, some matings of G0 adults produced light brown and white eggs (Supplementary Table 2). Individual fertilized G1 eggs carrying a lethal mutation cannot be distinguished from unfertilized eggs (which permanently remain white); but usually most or none of a female’s eggs are fertilized. Thus the variable ratios of white eggs among some G1 families are likely due to homozygous lethal effects of the *white* mutations. To determine whether lethality due to off-target effects on other essential genes could also occur, we identified two genes in the *H. armigera* genome with regions identical to the targetted region of *white*, but we detected no mutations at these sites by sequencing DNA of mosaic G0 individuals (Supplementary Table 3 and Supplementary Table 4, Supplementary Figure S5A,B). We cannot rule out a certain proportion of lethality due to other off-target effects, but these would have to have occurred in the germ-line of both G0 parents independently. We conclude that frame-shifting mutations of *white* that prevent protein expression are recessive embryonic lethal in *H. armigera*.

One cross of G0 adults produced some G1 eggs that developed light brown pigment more slowly than the control, and produced less pigment overall. These eggs also hatched with significantly lower frequency. The larvae from these eggs developed the light brown epidermal stripes more slowly, and in the later instars the larvae showed further unusual phenotypes such as a double head due to retention of the previous head capsule after a larval molt, large head size and moisture exuding from the larval skin (Supplementary Figure S6A–E). More than 10 percent of larvae had a double head in the third instar (Supplementary Figure S6A). We sequenced the targeted site of *white* in these mutants, which revealed a deletion of three nucleotides CAT, maintaining the reading frame but removing the isoleucine at position 119 (GenBank Accession KU754477, Supplementary Figure S6F). We named this mutant *W*^*I-119*^. Larvae with unusual phenotypes were heterozygous +/*W*^*I-119*^. No homozygous *W*^*I-119*^/*W*^*I-119*^ larvae were recovered from this mating or any other subsequent mating from the line developed from this family. We conclude that the *W*^*I-119*^ mutant allele is homozygous recessive lethal, and has dominant effects evident in heterozygotes. Some heterozygotes survived to adulthood (Supplementary Figure S6H) and produced progeny. The heterozygous status of G3 larvae with the double head phenotype was further confirmed with the Surveyor nuclease assay (Supplementary Figure S6G).

**Figure 2:**
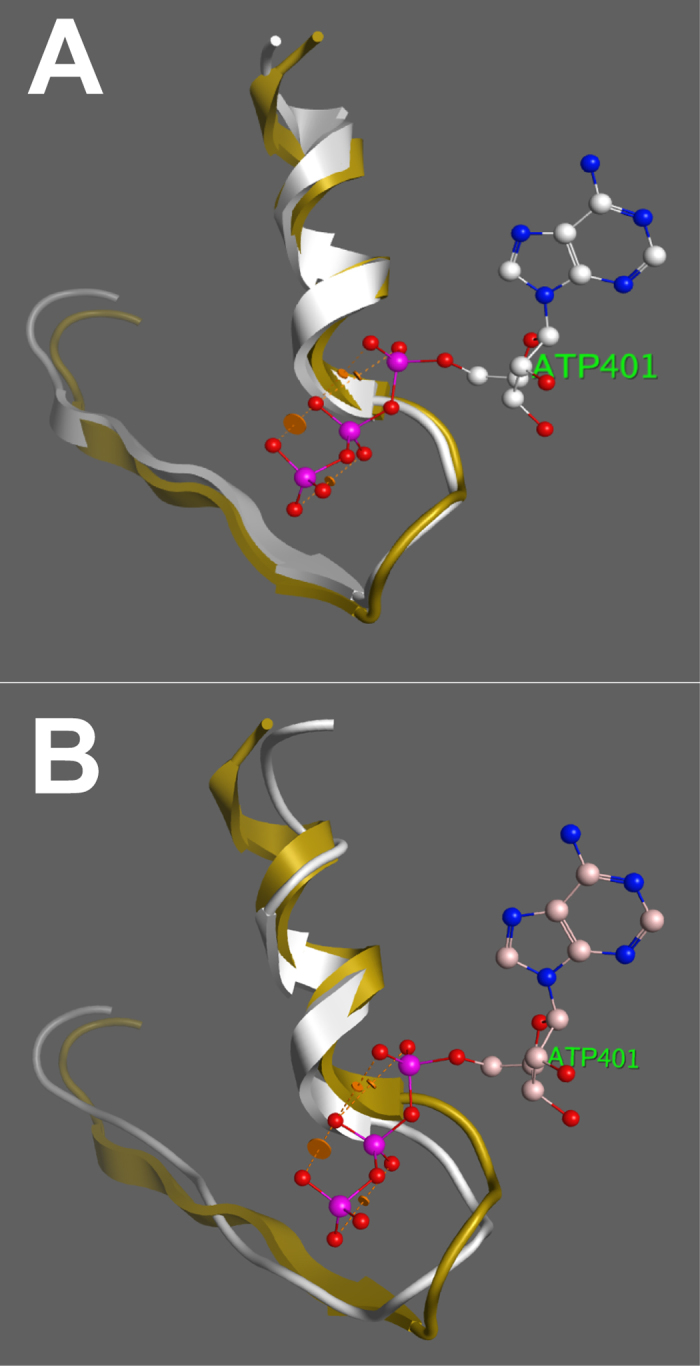
Partial homology models of wild type White and Ha-W^*I-119*^proteins with ATP. **(A)** Amino acids 113-135 of the White protein are shown in white, and the corresponding residues of the reference model are shown in gold. (**B**) The corresponding region of the W^*I-119*^protein is shown in white. Deletion of Ile-119 disrupts the β-sheet and pulls the loop away from the ATP. 3FVQ of the FbpC ABC transporter from *Neisseria gonorrhoeae* was used as reference model.

An isoleucine or valine at position 119 is highly conserved across insect *white* genes, and occurs in the middle of a β-strand directly adjacent to a highly conserved ATP binding domain (Supplementary Figure S7). No insect *white* gene to our knowledge has a gap in this region. To examine the possible functional consequences of deletion of Ile-119, we performed homology modelling of the wild-type White protein and the mutant W^*I-119*^ protein (Supplementary Figure S8). The loop of the *H. armigera* White protein near the phosphates of ATP (white ribbons in Fig. 2A showing amino acids 113–135) corresponds closely to that of the superimposed template (gold in Fig. 2A). Deletion of Ile-119 in the mutant W^*I-119*^ protein (white in Fig. 2B) disrupts the β-strand and deforms the loop relative to the template (gold in Fig. 2B). We predict that ABC heterodimers containing the mutant W^*I-119*^ protein are not able to transport pigment precursors because ATP binding and/or hydrolysis is prevented. This inability is lethal to *W*^*I-119*^/*W*^*I-119*^ homozygotes, similar to frame-shifting mutants that prevent expression of any White protein. However, production of mutant W^*I-119*^ protein in heterozygotes competes with the wild-type White protein in forming heterodimers with Scarlet, Brown and Ok, thus producing a partially dominant negative phenotype by reducing the overall concentration of active transporters. All the other *white* mutations causing frameshifts do not produce such competing proteins, and are therefore recessive.

### Induced mutations in the *scarlet* gene in *H. armigera*

**Figure 3:**
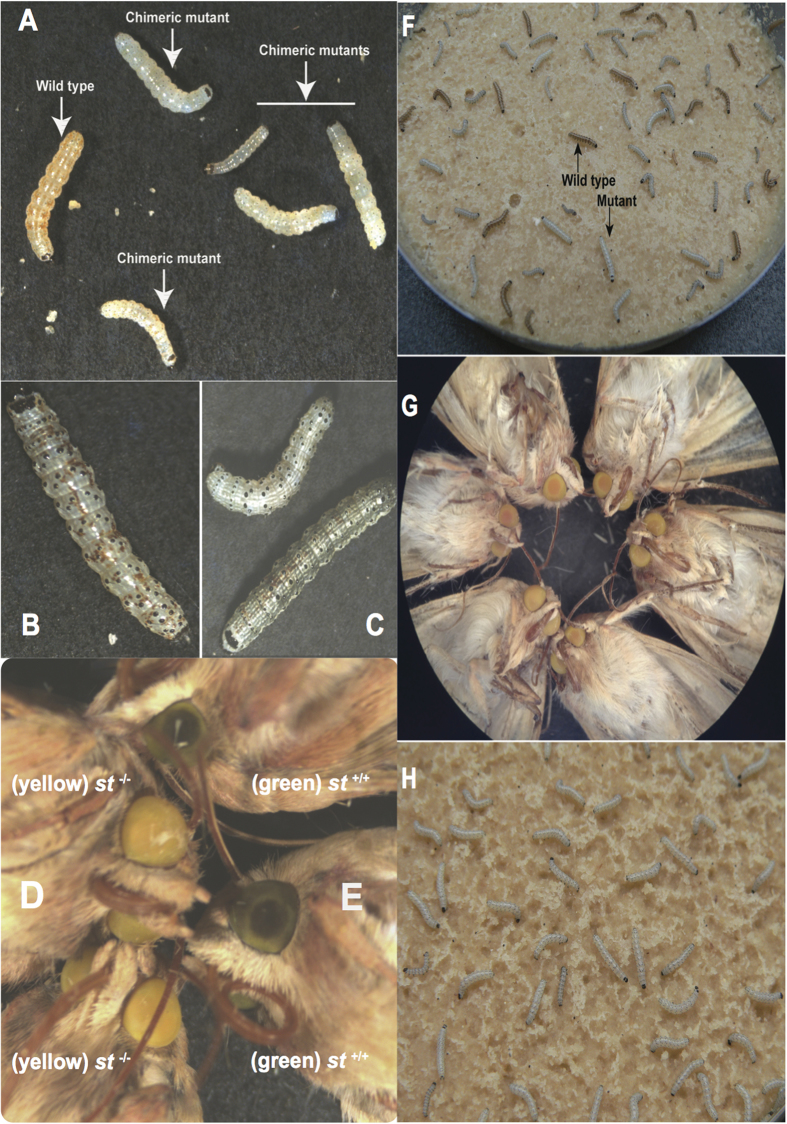
CRISPR/Cas9 induced mutations at the *scarlet* locus in G0 individuals and isolation of homozygous mutants in the G1 and G2 generations. (**A**) Control larvae and *st* mutant first instar larvae (48 hours post hatching). (**B**) *Ha-st* mutant (4^th^ instar) mosaic larvae in G0. (**C**) Fourth instar G0 progeny with more than 90% of somatic cells mutated. (**D**) Yellow eye of *scarlet* mutant adults. (**E**) Wild type *H. armigera* adults with green eyes. (**F**) Phenotypes of G1 heterozygous and homozygous *Ha-st* mutant larvae at 3^rd^ instar. Light brown larvae are wild type and larvae with whitish/greenish cuticle are homozygous *scarlet* mutants. (**G**) Phenotypes of G1 homozygous mutant adults (yellow eye) developed from the G1 mutant whitish/greenish cuticle color larvae. (**H**) G2 larval phenotype developed from homozygous yellow eye color mutants.

We targeted exon 6 of the *H. armigera scarlet* gene (*Ha-st*, GenBank Accession KU754478, Supplementary Figure S1B) to induce mutations. Because of SNP polymorphisms in the target region, we used two different constructs for the guide RNA (Supplementary Table 1). Some injected eggs remained all or partially white, and produced chimeric larvae with clear patches within the stripes of light brown pigment (Fig. 3A,B). Second through fifth instar larvae had additional, brown and black pigments in the cuticle that appeared as in the wild type, but the mosaic pattern of light brown and clear remained over that ground pattern (Fig. 3C). Mosaic larvae developed to adulthood, and many adults showed mosaic patterns of yellow and green ommatidia. Matings of some mosaic adults produced viable progeny, which were segregating for presence or absence of light brown pigment in the first larval instar (Fig. 3F). Segregation ratios deviated slightly from 50% (Table 1), probably because of mosaicism in the germ-line. White larvae developed into adults with entirely yellow eyes (Fig. 3D) compared with wild-type green eyes (Fig. 3E). Crosses using yellow-eyed moths produced all white eggs (Supplementary Figure S2A), all completely white first-instar larvae (Fig. 3H), and 100% yellow adult eyes (Fig. 3G). Further crosses established that the white larval phenotype and yellow eyes were inherited as a single, recessive trait. Sequencing of genomic DNA from yellow-eyed G2 adults revealed insertion of two bases in the targeted site, causing a frameshift leading to premature truncation of the protein at residue 223 (Supplementary Figure S9). No other visible phenotype was affected, and viability of larvae, pupae and adults appeared to be normal.

In *Drosophila* and *Bombyx*, the heterodimer of Scarlet and White transports 3-hydroxykynurenine derived from tryptophan into pigment cells where it is processed to form the first ommochrome^38^,^39^,^40^. *Drosophila scarlet* mutants have red eyes because the import of pteridine precursors is unaffected, but *Bombyx scarlet* mutants (as well as *Bombyx white* mutants) have white eyes because pteridines do not contribute to eye color in that species^22^,^34^. There is evidently a yellow pigment remaining in the *scarlet* mutant eyes of *H. armigera*, which we attempted to identify (see below).

### Induced mutations in the *brown* gene in *H. armigera*

We targeted exon 2 of the *H. armigera brown* gene (*Ha-bw*, GenBank Accession KU754480, Supplementary Figure S1D and Supplementary Table 1) to induce mutations by CRISPR/Cas9. In contrast to the mosaic phenotypes observed in G0 individuals when targetting *w* and *st*, we did not observe any differences in eggs injected with the construct targetting *brown*, or in larvae hatching from them. Sequencing of genomic DNA from G0 larvae showed large deletions in the targeted site (Supplementary Figure 10A) but no larval or adult mosaic phenotypes were evident. All adults developing from G0 larvae had green eyes with no mosaicism, indistinguishable from wild type. Therefore, unlike *Drosophila* but similar to *Tribolium*, the brown gene does not appear to contribute to pigmentation in *H. armigera*.

### Induced mutations in the *ok* gene in *H. armigera*

**Figure 4:**
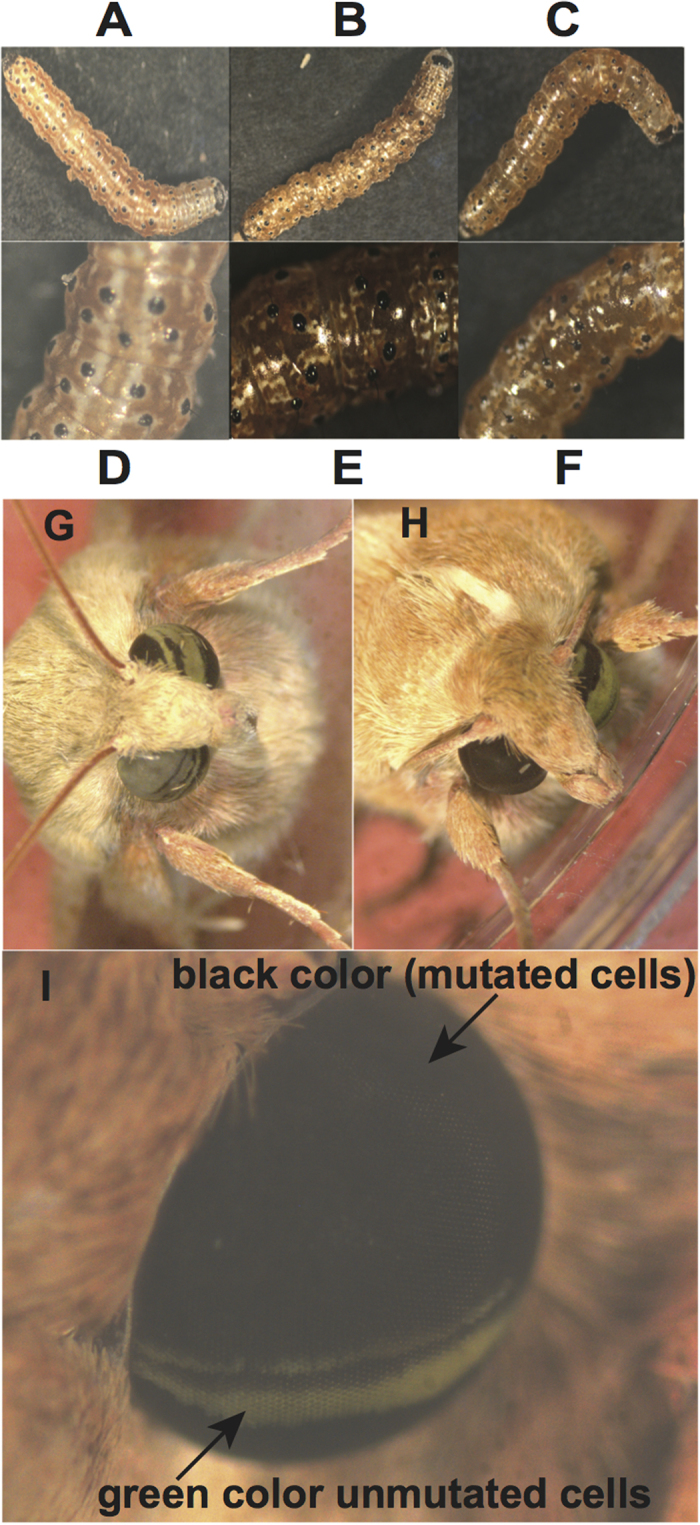
CRISPR/Cas9 induced mutations at the *ok* locus in G0 larvae and adults. (**A**) Control 3^rd^ instar wild type larval phenotype. (**B,C**) Two chimeric mutated 3^rd^ instar larvae with different ranges of somatic cell mutation and mosaic skin pattern. (**D–F**) Magnified images of the same larvae in (**A–C**). (**G,H**) Two mosaic mutated adults developed from the G0 injected larvae which showed altered cuticle phenotypes. (**I**) Magnified images of the mosaic eye from the G0 adults with mutated and un-mutated eye cells. More than 95% cells are mutated, demonstrating the high efficiency of CRISPR against the *Ha-ok* locus.

It was recently reported that a paralog of *brown* is present in *B. mori,* which is named *ok*^23^. These two genes occur in a tail-to-tail tandem array in the *Bombyx* genome, and are likely to have arisen from a gene duplication in an ancestral lepidopteran. The *ok* gene is also present in *H. armigera* (GenBank Accession KU754481, KU754482) and we targeted exon 2 (Supplementary Figure S1C and Supplementary Table 1) to generate mutants. Injected eggs appeared normal, but some produced larvae with a mosaic pattern with clear patches extending across the white stripes, indicating an absence of uric acid (Fig. 4A–F). Some G0 adults had chimeric eyes with alternating stripes of green and black ommatidia (Fig. 4G–I).

**Figure 5:**
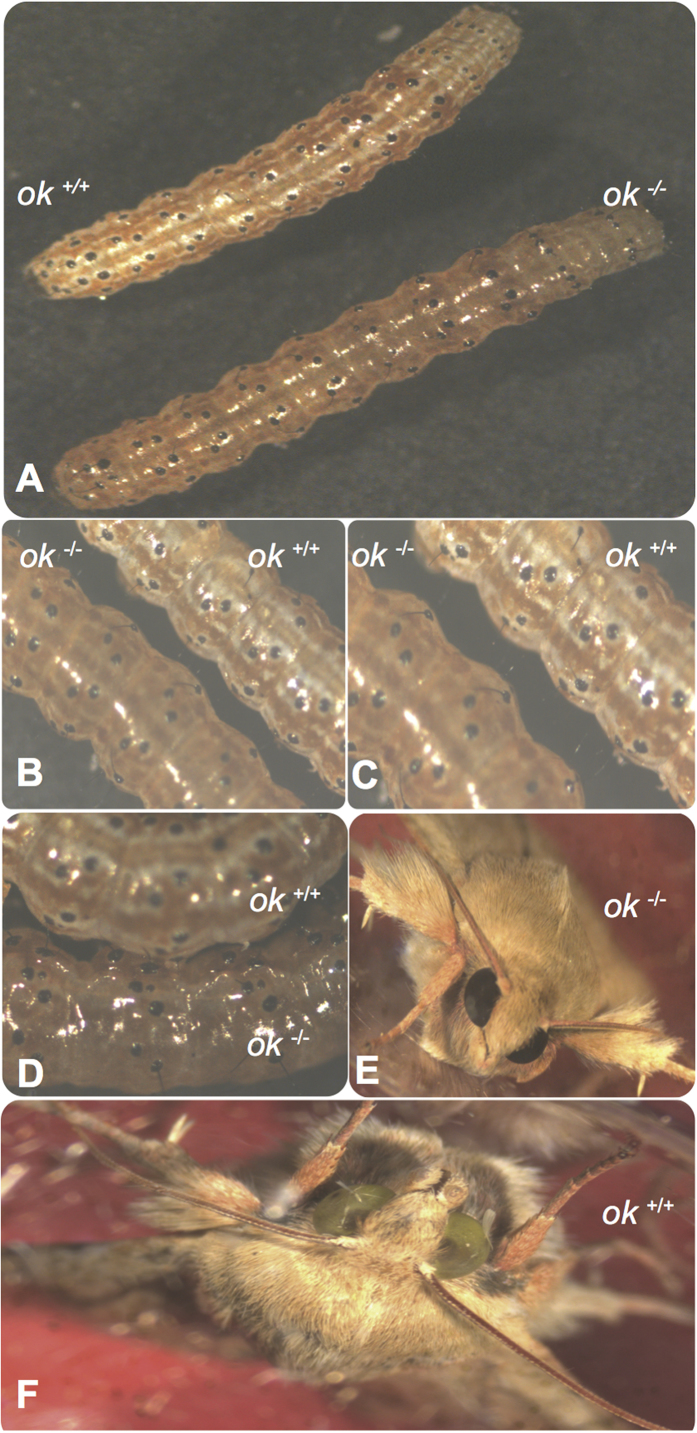
Germ-line transmission of the *Ha-ok* gene mutation in homozygous G1 progeny. (**A**) Epidermal phenotypes show *Ha-ok* mutant individuals (*ok*^−/−^) lack of uric acid accumulation and the wild type (WT) phenotype (*ok*^*+/+*^) with opaque cuticle. (**B,C**) Magnified images of mutant (*ok*^−/−^) and wild type (*ok*^*+/+*^) larvae at 3^rd^ instar. (**D**) Magnified translucent phenotype of mutant larva (*ok*^−/−^), compared to control larva (*ok*^*+/+*^). (**E**) Homozygous G1 adult developing from the G1 larvae has black eyes. (**F**) Wild-type moths with green eyes.

G0 moths were fertile, and sib matings produced larvae with no white stripes (Fig. 5A–D). The stripes instead had a translucent, oily appearance similar to *ok* mutants in *Bombyx*, due to the absence of uric acid^23^. Adults developing from these larvae had eyes that were completely black (Fig. 5E,F), but produced eggs with normal brown pigmentation (Supplementary Figure S2B). Sequencing of genomic DNA of individuals of one G2 family with black eyes revealed four different mutant alleles with insertions or deletions in the targeted area. (GenBank Accessions KU754483–KU754490, Supplementary Figure S10B,C).

### Identification of pigments using double mutants

**Figure 6:**
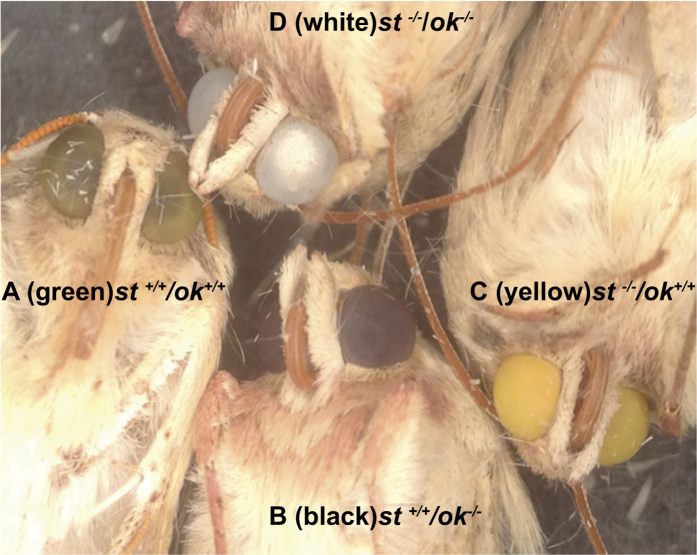
Single and double mutants for *scarlet* and *ok*. (**A**) Wild type *H. armigera* has functional wild type *scarlet* and *ok* genes and green eyes. (**B**) Homozygous mutant for *ok* with black eyes. (**C**) Homozygous mutant for *Ha-st*^−/−^ with yellow eyes. (**D**) Double mutant (*Ha-st*^−/−^ and *Ha-ok*^−/−^) with white eyes.

To our knowledge, the Ok protein has not yet been suggested as a transporter for eye color pigment precursors in insects. We hypothesized that in *H. armigera*, Scarlet and Ok each heterodimerize with White to form transporters for ommochromes and pteridines respectively, and the combination of these two pigments provides the green color in the adult eye. To investigate this hypothesis we crossed the *st/st* with *ok/ok* mutants. F_1_ hybrids all had wild-type larval pigmentation and adult eye color, showing that these two genes do not complement each other with respect to any of the phenotypes observed. In the F_2_, two types of larvae were observed: 1) larvae with wild-type cuticles, predicted to be +/+ or +/*st* for *scarlet* and +/+, +/*ok* or *ok/ok* for *ok*, which developed into moths with either green (Fig. 6A) or black (Fig. 6B) eyes, and 2) larvae with white cuticles, predicted to be *st/st* for *scarlet* and +/+, +/*ok* or *ok/ok* for *ok*, which developed into moths with either yellow (Fig. 6C) or white (Fig. 6D) eyes. The white-eyed moths proved to be *st/st ok/ok* double mutants, as all of their progeny and grandprogeny were white as eggs (Supplementary Figure S2D) and as larvae, and had white eyes as adults.

**Figure 7:**
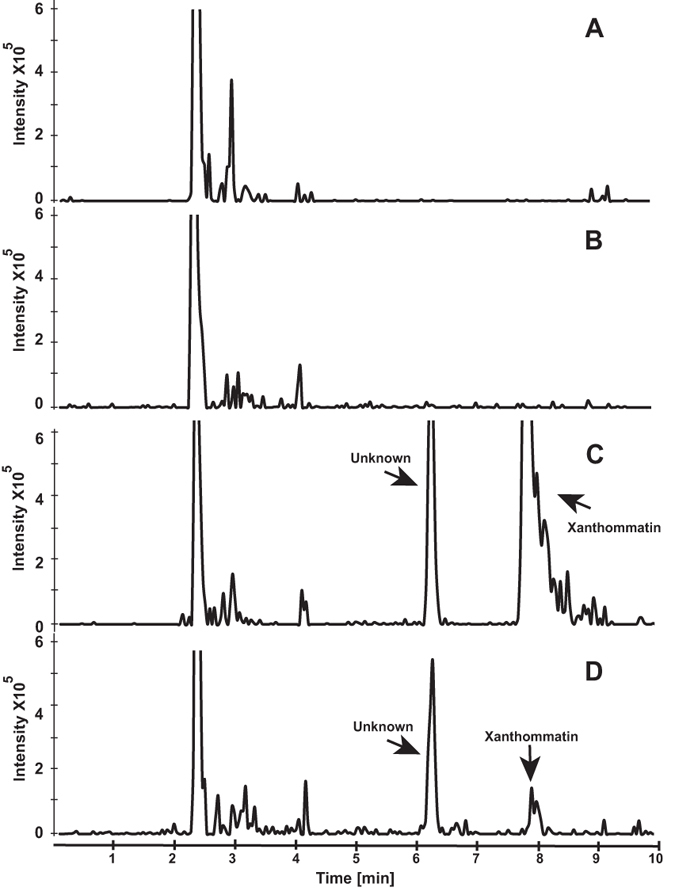
LC-MS chromatograms (extracted ion chromatograms in positive mode) of extracts of insect eyes for 4 different insect lines. (**A**) Yellow-eyed *scarlet* mutants. (**B**) White-eyed *scarlet* and *ok* double mutant. (**C**) Black-eyed *ok* mutant. (**D**) Wild type.

**Figure 8:**
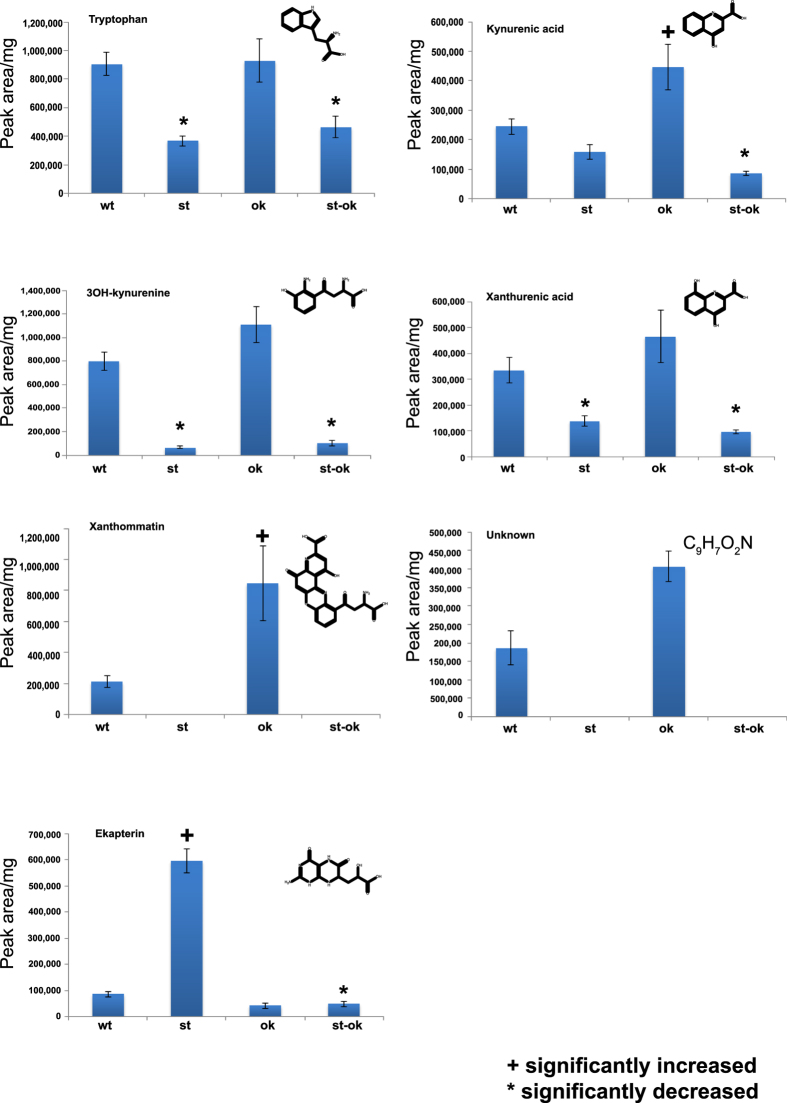
Distribution of pigments in eyes of single (*st, ok*) and double (*st-ok*) mutants and wild type (wt). Error bars represent standard errors of the means. Statistical tests are described in the Methods.

To identify the pigments responsible for the yellow eye of the *H. armigera st/st* mutant and the green eye of the wild type, we isolated small molecules from the eyes of wild type (green), *st/st* homozygotes (yellow), *ok/ok* homozygotes (black) and *st/st ok/ok* double mutants (white) and analyzed the extracts by LC-ESI-MS. The ommochrome xanthommatin was not detectible in yellow or white eyes, but was present in green eyes and even more abundant in black eyes (Fig. 7). Compounds with significantly different concentrations among the four genotypes are shown in Fig. 8. Tryptophan and its metabolites kynurenic acid, 3-hydroxy kynurenine, and xanthurenic acid, precursors of ommochromes, occur at significantly lower concentrations in eyes of both types of *st/st* homozygotes (Fig. 8). An accurate molecular mass of 162.0547 (positive mode) could be determined for an unknown compound having the same distribution as xanthommatin. (Figs 7 and 8). This yielded a unique empirical formula that is consistent with a variety of compounds, all smaller than typical ommochromes. Comparison with authentic standards ruled out 2,8-quinoline diol, indol-3-carboxylic acid, indol-2-carboxylic acid, and indol-5-carboxylic acid, but the structure remains to be determined. On the other hand, the pteridine ekapterin^41^ (Supplementary Figure S11) is greatly elevated in the yellow eyes of the *st/st* homozygotes that retain functional White and Ok proteins. This same heterodimer transports uric acid which is also derived from guanine into pigment granules in the larval epidermis.

## Discussion

We have used CRISPR/Cas9-based mutagenesis to experimentally inactivate genes encoding ABC transporters that have been implicated in insect pigmentation, largely from previous studies of spontaneously occurring mutations in model systems. The CRISPR/Cas9 system offers several advantages over previous approaches. Somatic mosaic effects can be detected for mutations that are otherwise embryonic lethal and for which a pigmentation phenotype could not be scored in a naturally-occurring mutation in the germ-line, as we discovered for the *white* gene of *H. armigera*. The high efficiency of gene disruption ensures that pigmentation phenotypes will be observed if they are caused by gene inactivation; and conversely when no phenotype is observed we can be confident that the candidate plays no role in observable changes in pigmentation, as we discovered for the *brown* gene of *H. armigera*. When combined with the analysis of pigments in the eyes of single and double mutants, the approach can yield insights into how the balance of different heterodimers of these ABC half-transporters affects the differential distribution of ommochromes and pteridines in a species that makes use of both for eye pigments.

The embryonic lethality of *white* in this species was surprising, since white-eyed insects due to inactivating mutations in this gene are viable in many other species, including *D. melanogaster, B.mori, T. castaneum*, and *A. gambiae*, and are even used as recipients for genetic transformation using the wild-type *white* gene as a marker. White has a demonstrated role in tryptophan, guanine and uric acid transport relating to wild eye color and cuticle tanning^17^,^34^. Other phenotypes of *white* mutations have been reported; including reduced sexual stimulation in *Drosophila*^42^ and different responses to anaesthetics^43^, but we are unaware of previous reports of lethality. Unlike larvae of the species mentioned, caterpillars of *H. armigera* incorporate both ommochromes and urate into epidermal pigment granules; and *white* mutants that are unable to sequester their precursors from the hemolymph may suffer from their toxic effects. For example, *Anopheles* mosquitoes induce the enzyme 3-hydroxykynurenine transaminase to detoxify 3-hydroxykynurenine that accumulates due to the breakdown of tryptophan after a blood meal, and the *Plasmodium* parasite uses the further metabolite xanthurenic acid as a dependable cue to initiate gametogenesis^44^. However, the double mutant *st/st ok/ok* is viable, even though both ommochrome and urate sequestration pathways are knocked out. This points to a vital role of the White protein in embryonic development of *H. armigera* that remains to be elucidated. Moreover, phenotypes of larvae heterozygous for the deletion of Ile-119 point to other functions in larval development that have previously not been attributed to *white*.

*Drosophila* eyes contain both ommochromes and pteridines, and *scarlet* mutations in that species block the uptake of ommochromes and shift the balance toward the red pteridines. Correspondingly, *brown* mutations block the update of pteridines and shift the balance in the eye towards the brown ommochromes. However, *brown* does not seem to have a similar effect in the Lepidoptera or Coleoptera that have been investigated so far. RNAi directed against *brown* in *Tribolium* had no visible effect on eye pigmentation^1^. No spontaneous mutations of *brown* affecting eye color have been discovered in *Bombyx*; any such mutations should map very close to the *ok* mutations responsible for the oily skin phenotype since *ok* and *brown* are adjacent in the Bombyx genome. The paralogous gene *ok* found in Lepidoptera may have taken over the function of *brown* in eye pigmentation, yet full-length copies of *brown* encoding apparently intact protein sequences are present in the *H. armigera* and *Bombyx* genomes. The function of Brown in these systems remains to be elucidated.

The phenotypes we have observed shed light on the how the balance between the various heterodimers involving the White protein affects patterns of pigmentation. The deletion of a single amino acid allows the production of an otherwise full-length White protein, that apparently competes with the wild-type White protein for Scarlet and Ok partners. This probably reduces the concentration of functional transporters of both types by half, causing abnormalities but not lethality. Scarlet and Ok also evidently compete for White partners; the knockout of one of them appears to increase the heterodimers produced by the other, leading to elevated concentrations of pteridines or ommochromes respectively in the eye, compared with the wild type. The function of OK in *H. armigera* appears to be analogous to that of Brown in *Drosophila* in this regard.

The identities of several of the ommochromes and pteridines involved in larval and adult eye pigmentation remain to be determined. Our results indicate that the ommochrome xanthommatin and the pteridine ekapterin are major pigments in the *H. armigera* eye. The mechanism of xanthommatin formation in *Drosophila* is still controversial; claims have been made for an enzyme, phenoxazinone synthetase, but this has never been isolated from *Drosophila*^6^; and xanthommatin can also be formed by spontaneous condensation of 3-hydroxykynurenine. The biochemical pathway leading to ekapterin is unknown. The differential distribution of eye pigments among different CRISPR/Cas9-induced mutants presented here will be useful in elucidating their biosynthetic pathways in the future.

## Materials and Methods

In this study, we used the TWB3 strain of *H. armigera* originally collected from the vicinity of Toowoomba, Queensland, Australia. The insects were reared in the laboratory for continuous supply for several generations using single pair crosses to minimize inbreeding. The larvae of *H. armigera* were hatched from eggs, and neonates were reared on artificial diet (Bio-Serv, Frenchtown, NJ, USA) at 26 °C with 16-h light/8-h dark^45^.

### Target design and *in vitro* synthesis of sgRNAs and Cas9- coding mRNA

Cas9 nuclease capped mRNA was synthesized *in vitro* from the pMLM3613 plasmid^37^ (provided by Addgene), and the *Pme*I digested plasmid was used as a template for transcription. The plasmid has the T7 promoter upstream of Cas9 coding sequences. The mMESSAGE mMACHINE T7 ULTRA kit (Life Technologies) was used to synthesize the Cas9 mRNA and add a poly(A) tail following the manufacturers’ instructions. Following ethanol precipitation, mRNA was suspended in an appropriate volume of water to achieve a final concentration of 200–300 ng/μl, distributed in small aliquots and stored at −80 °C for future use. Four *H. armigera* genes of half-ATP binding cassette (ABC) transporters—*white, scarlet, brown* and *ok*—were used in this study. At least one pair of oligonucleotides against each gene was designed, using the ZiFit Targeter version 4.2 website (zifit.partners.org)^46^,^47^ to identify potential target sites for our studies and cloned into the pDR274 plasmid^37^ provided by Addgene. The genomic target site sequences used in this study are listed in Supplementary Table 1. Plasmid pDR274 harboring a T7 promoter upstream of cRNA and guide RNA was digested with *Bsa*I. The annealed oligonucleotides with overhangs that were compatible with directional cloning into the *Bsa*I digested pDR274 vector were cloned. The newly constructed sgRNA plasmids were denoted Sh-Haw, Sh-Hast, Sh-Habw and Sh-Haok. Each plasmid was digested with *Dra*I to linearize it, and the linearized plasmids were used to transcribe *in-vitro* the guide RNA using the MAXIscript T7 kit (Life Technologies) following the manufacturers’ instructions. The sgRNAs were then purified by either LiCl or ammonium acetate precipitation and re-dissolved in RNase free water. To avoid multiple freeze-thawings, the RNAs were stored in aliquots at −80 °C.

### Microinjection of Cas9 and sgRNA RNAs into *H. armigera* eggs

Embryos of the TWB3 strain of *H. armigera* were used for microinjection. Fertilized eggs were collected within one hour of oviposition. The eggs were lined up on double sided adhesive tape attached to a microscope slide after treatment with 0.5% bleach for 20 seconds and followed by frequent washing with distilled water to collect the eggs from muslin cloth. A solution containing mRNA ~200 ng/μl and sgRNA ~25 ng/μl was back loaded into homemade glass needles and injected into each egg. Each embryo was injected with approximately 2 nl of solution containing sgRNA and Cas9 mRNA. The injected eggs were incubated at 25 °C until hatching. Injected embryos were checked under the light microscope within 24 hours of injection. Only embryos that developed normally were used for further analysis. The G0 animals were inspected for any type of phenotypes in the following days, during different larval, pupae and adult stages.

### Analysis of germ-line mutation frequency in targeted loci

Crossing was used to test whether mutations in injected G0 could be transmitted to the G1 offspring, and also to calculate the probability of inheritance. Mosaic individuals for each locus were crossed with each other to quickly acquire G1 offspring whose alleles were both mutated but at different locations (compound heterozygotes). To calculate the inheritance efficiency of Cas9/sgRNA-mediated gene alteration in G1 progeny for the *Ha-st* locus, the change of skin color was used as a marker for mutation in G1 offspring.

### Analysis of mutations using sequencing and Surveyor nucleases

Primers were designed flanking the target sites for each gene and used to PCR amplify products from mosaic individuals. PCR amplicons were TA-cloned and sequenced to determine the exact mutation type. To detect induced mutations in G3 larvae, we extracted genomic DNA of larvae at 4^th^ instar, amplified a region of 257 bp containing the *Ha-w* target site, and used the amplicons for the Surveyor nuclease assays. In this assay, the T7 endonuclease recognizes base substitutions and small insertions/deletions in a DNA duplex, and cleaves the 3′end side of the mismatched sites in both DNA strands. The resulting size difference is visualized by agarose gel electrophoresis. To allow complementary but mismatched strands to anneal, PCR products (10 μl) were incubated at 95 °C for 5 min, then the temperature gradually ramped down from 95 °C to 85 °C at 2 °C s^−1^ and from 85 °C to 25 °C at 0.1 °C s^−1^. T7 Endonuclease (New England Biolabs^®^ Inc.) was then added and samples were incubated at 37 °C for 30 min to digest the annealed PCR products at the sites of mismatch. Nuclease digested PCR products were analyzed by agarose gel electrophoresis, and sequenced at the Max Planck Institute for Chemical Ecology, and analyzed using Sequencher software (GeneCodes).

### Homology identification, protein alignments and phylogeny of half-ABC transporters in *H. armigera*

Amino acid based sequences from *Bomby mori* were used as queries to BLAST search our in-house *H. armigera* transcriptome and genomic DNA data. Contigs similar to four homologues of half ATP transporters were retrieved and named as Ha-White, Ha-Scarlet, Ha-Brown and Ha-OK half ABC transporters. The genes were characterized for intron/ exon structure. Half ABC transporter sequences from *H. armigera* and publicly available genomes of other species were used for blast search and similarity comparison. The maximum likelihood phylogenetic tree was constructed in the MEGA program, version 6^48^, using default parameters in all categories except the following: bootstrapping with 500 replicates, LG model of amino acids substitution with Gamma distributed substitution rates with invariant sites, based on best model determination within the MEGA program and partial deletion treatment of gaps/missing data^49^.

### Metabolite analysis by LC- ESI-IonTrap-MS

Insect eye samples of around 10 mg were extracted with 0.5 mL of methanol. The samples were homogenized with the help of 3 mm stainless steel balls and a paint shaker (Skandex SO-10M, Fluid Management Europe, The Netherlands). After centrifugation an aliquot of the supernatant was analyzed by LC-ESI-IonTrap-MS using a Bruker Esquire 6000 ion trap mass spectrometer (Bruker Daltonics, Bremen, Germany) operated in alternating ionization mode (electrospray ionization) in the range m/z 60–1,000 (Skimmer voltage, 53 eV; capillary exit voltage, −117.3 eV; capillary voltage, 4,000 V; nebulizer pressure, 35 psi; drying gas, 11 l min −1; gas temperature, 330 °C) coupled to an Agilent 1100 series HPLC (Agilent Technologies, Waldbronn, Germany). Elution was accomplished using a Nucleodur Sphinx RP column (250 × 4.6 mm, 5 um; Macherey-Nagel, Duren, Germany). Mobile phases were 0.2% formic acid (v:v) (A) and acetonitrile (B), starting with 10% B, followed by a gradient to 90% B in 20 min, the column was washed with 100% B for 3 min and equilibrated for 4 min at 10%B. The molecular ion peaks [M+H]^+^ of the analytes were monitored in positive mode (extracted ion chromatograms), and the area under the peak was used for relative quantification. The peak area for each compound was normalized by the sample weight for each sample to obtain relative sample concentrations in peak area/mg of weight.

### Identification of Ekapterin

Low resolution ESI mass spectra and MS^2^ and MS^3^ spectra of ekapterin were obtained using a Bruker Esquire6000 Ion Trap Mass spectrometer (Bruker Daltonics, Bremen, Germany) in positive and negative ionization mode, respectively (Supplementary Figure S11A,B). LR-ESI-MS: m/z 270.1 [M+H]^+^ and m/z 268.1 [M−H]^−^ High resolution ESI mass spectra were recorded on an Orbitrap XL mass spectrometer (Thermo Fisher Scientific, Bremen, Germany). The molecular formula of ekapterin was determined to be C_9_H_11_O_5_N_5_ (calcd. monoisotopic mass 270.0833) from the [M+H]^+^ ion peak at m/z 270.0831 in HRESIMS.

### Statistical analyses of metabolites

In order to test whether *H. armigera* strains differed in their content of certain metabolites the method of generalized least squares (gls, nlme library of the R computer program) was used^50^. The VarIdent variance structure (which allows each *H. armigera* strain to have a different variance) was applied. P-values were obtained by removing the explanatory variable and comparison of models with a likelihood ratio test^51^. In order to find out whether transformed *H. armigera* strains were different to the wild type *H. armigera*, factor level reduction was applied. All analyses were done in R 3.2.0^55^.

### Homology based modeling of White (Ha-W) and Mutant W^*I-119*^ protein

Before modeling of White and mutant (W^*I-119*^), identification of a suitable homology model was done by using the protein blast search capability of MOE 2014.09^53^. This search identified the structure 3FVQ of the FbpC ABC transporter from *Neisseria gonorrhoeae* as a homologue of the target sequence^54^. Using MOE 2014.09, we built the homology models for the wild type white (W) protein and the mutant white protein (W^*I-119*^), using the FbpC ABC transporter as a template, and compared them by superimposing the FbpC experimental crystal model on the homology models.

### Determination of ATP binding site

The White protein is a half-ABC transporter, which binds to ATP and uses ATP for active transportation of other molecules. The White protein homology model was used to determine the ATP binding domain in the wild type White protein and the mutant (W^*I-119*^) protein models based on the position of ATP in the crystal structure of 3FVQ.

### Potential off-target detection

The potential off-target candidate loci in the *H. armigera* genome for *white* gene crRNA were searched using the modified software TagScan Server modified from the Tagger program from Swiss Institute of Bioinformatics^55^,^56^ to evaluate the off targeting of the CRISPR/Cas9 system in *H. armigera*. Specific primers were designed across the potential off-target sites and DNA sequencing was performed for the evaluation of the off-target effects.

## Additional Information

**Accession codes:** The sequences reported in this paper have been deposited in the GenBank database (Accession Nos KU754476 to KU754490).

**How to cite this article**: Khan, S. A. *et al*. Functional analysis of the ABCs of eye color in *Helicoverpa armigera* with CRISPR/Cas9-induced mutations. *Sci. Rep.* **7**, 40025; doi: 10.1038/srep40025 (2017).

**Publisher's note:** Springer Nature remains neutral with regard to jurisdictional claims in published maps and institutional affiliations.

## Supplementary Material

Supplementary Information

## Figures and Tables

**Table 1:** Heritable genome editing with CRISPR/Cas9 for the *Ha-st* locus.

Cross	Pigmented larvae	Unpigmented larvae	Mutation
Family 1	109	101	48,09%
Family 2	90	90	50%
Family 3	58	100	63,29%
Family 4	90	150	62,5%

Offspring of four families of G0 adults are shown. Deviations from Mendelian segregation are due to mosaicism in the germ cells.
