# Supplementary material for: Functional analysis of the ABCs of eye color in Helicoverpa armigera with CRISPR/Cas9-induced mutations
Source: Sci Rep. 2017 Jan 5;7:40025. doi: 10.1038/srep40025 (PMC5214861; doi:10.1038/srep40025)
Supplement: Supplementary Information [file srep40025-s1.pdf]

Functional analysis of the ABCs of eye color in *Helicoverpa armigera* with CRISPR/Cas9-induced mutations.

Sher Afzal Khan, Michael Reichelt, and David G. Heckel

Supplementary Figure 1. Schematic representation of exons of ABC eye color transporter orthologs in *H. armigera*. (**A**) *Ha-w* (*white*) and (**B**) *Ha-st* (s*carlet)* gene exons are shown as boxes according to their exon size. (**C**) Alternative splicing variants of *ok* like gene in *H. armigera*, exon skipping type of splice variant, the last exon 12 in two splice variants is different from each other, are shown in green *Ha-ok-A* and red *Ha-ok-B*. (**D**) The *brown* gene's exon pattern in *H. armigera*, with target site locations in green and PAM sequences in red.

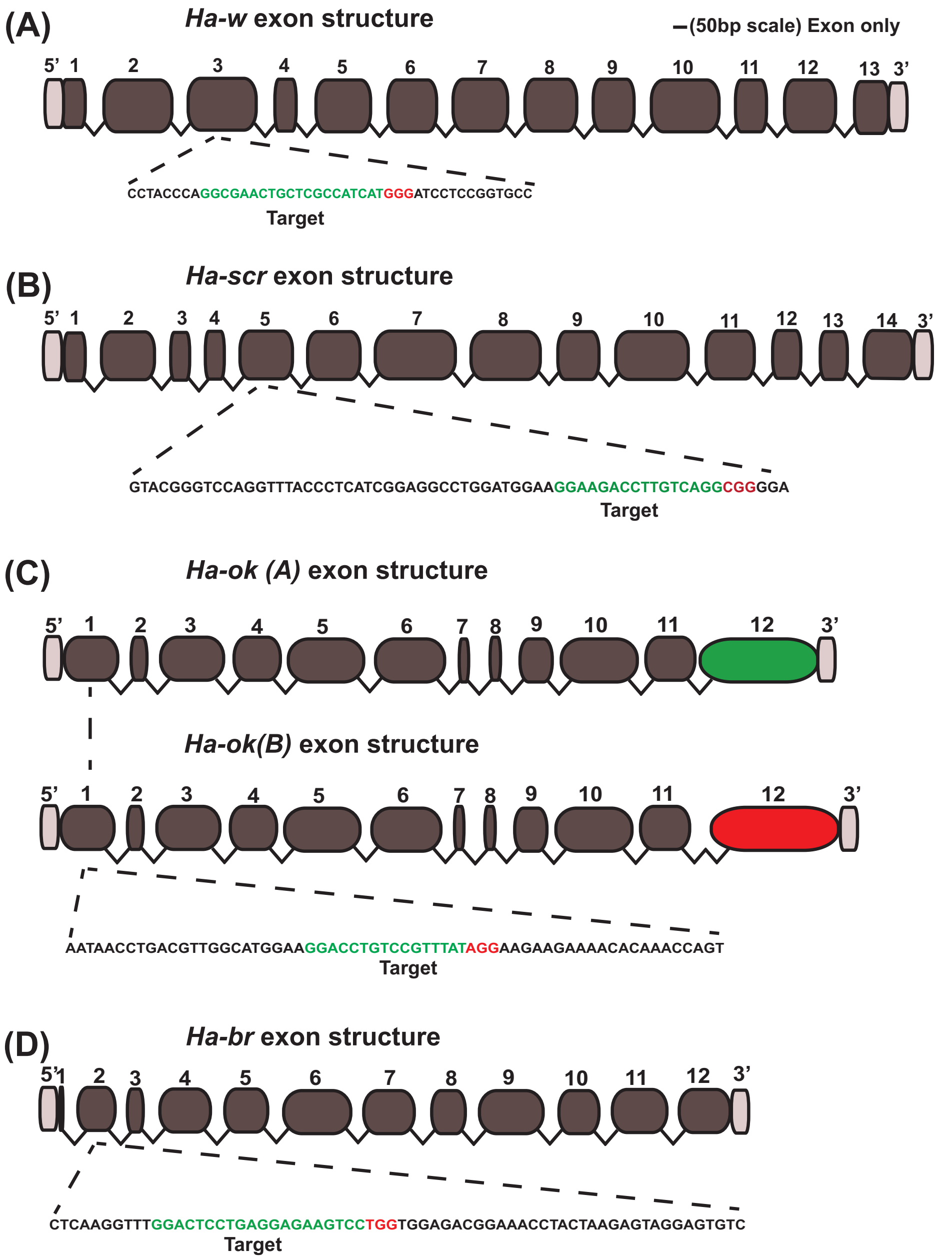
(A)
Ha-w exon structure
(50bp scale) Exon only
5' 1 2 3 4 5 6 7 8 9 10 11 12 13 3'
CCTACCCAGGCGAACTGCTCGCCATCATGGGATCCTCCGGTGCC
Target
(B)
Ha-scr exon structure
5' 1 2 3 4 5 6 7 8 9 10 11 12 13 14 3'
GTACGGGTCCAGGTTTACCCTCATCGGAGGCCTGGATGGAAGGAAGACCTTGTCAGGCGGGGA
Target
(C)
Ha-ok (A) exon structure
5' 1 2 3 4 5 6 7 8 9 10 11 12 3'
Ha-ok(B) exon structure
5' 1 2 3 4 5 6 7 8 9 10 11 12 3'
AATAACCTGACGTTGGCATGGAAGGACCTGTCCGTTTATAGGAAGAAGAAAACACAAACCAGT
Target
(D)
Ha-br exon structure
5' 1 2 3 4 5 6 7 8 9 10 11 12 3'
CTCAAGGTTTGGACTCCTGAGGAGAAGTCCTGGTGGAGACGGAAACCTACTAAGAGTAGGAGTGTC
Target

Supplementary Figure 2. Egg phenotypes of mono and double mutants display impairment for pigment accumulation. (**A**) Ommochrome granules are not formed in egg serosa cells from homozygous yellow eye mutants (*Ha-st*), even though embryos completely developed. (**B**) Eggs from homozygous *Ha-ok* mutant individuals. The eggs developed the normal brown color within 48 hours of egg laying. The ommochrome granules are formed in egg serosa cells as usual, although the pteridine pigment synthesis is impaired. (**C**) Eggs from control TWB-3 moths, photo taken within 48 hours eggs laying. (**D**) White eggs from double mutant individuals, lacking pigment accumulation.

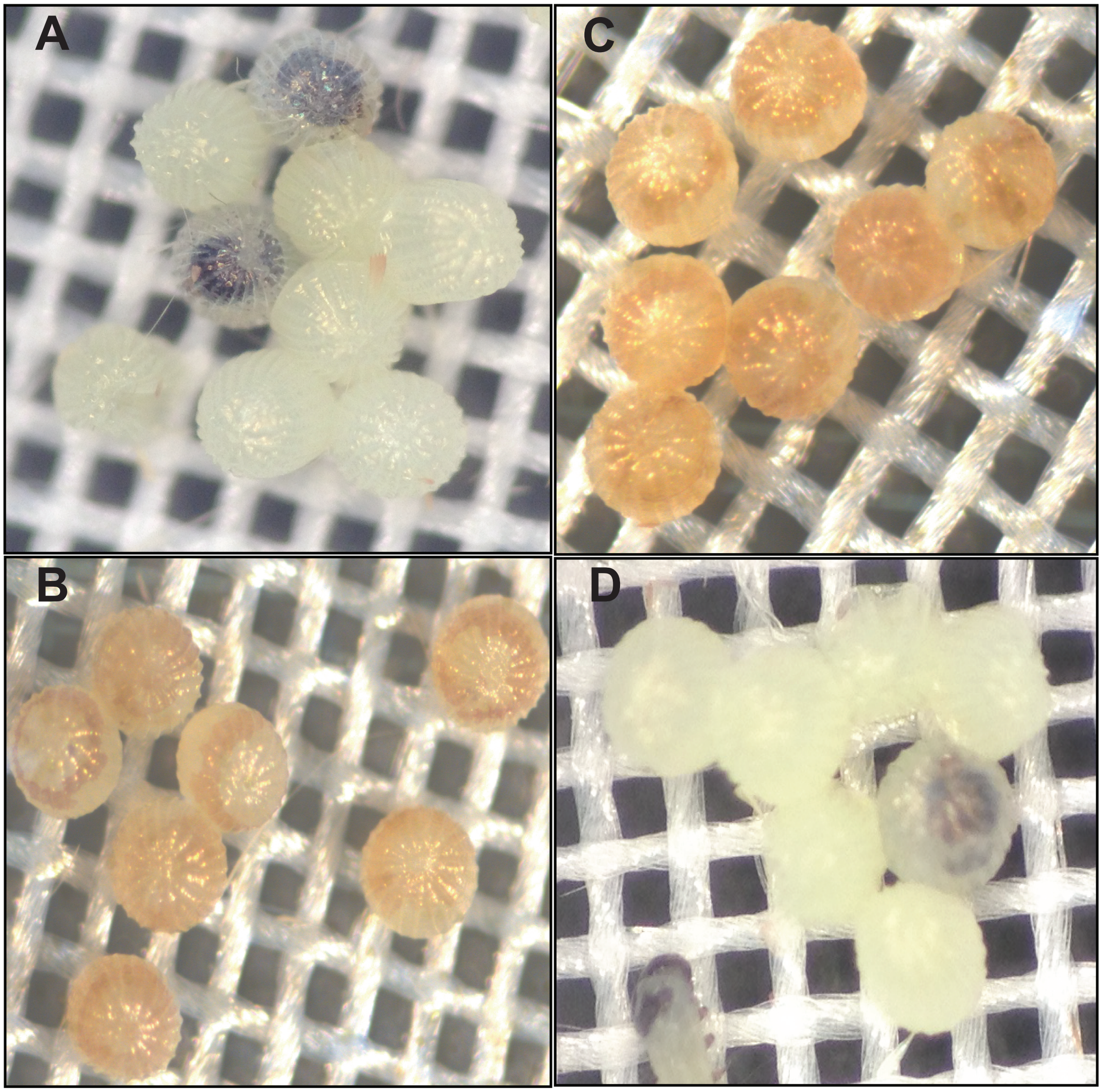

Supplementary Figure 3. CRISPR/Cas9-induced mutations at the *white* locus in G0 *H. armigera* larvae. (**A-D**) *Ha-w* chimeric mutants at 4th instar G0 larvae developed from different independent events. Each larva has a different pattern of somatic cell mutations. (**E-F**) Wild type 4th instar larvae have wild type phenotypes. The scale bar is 1mm.

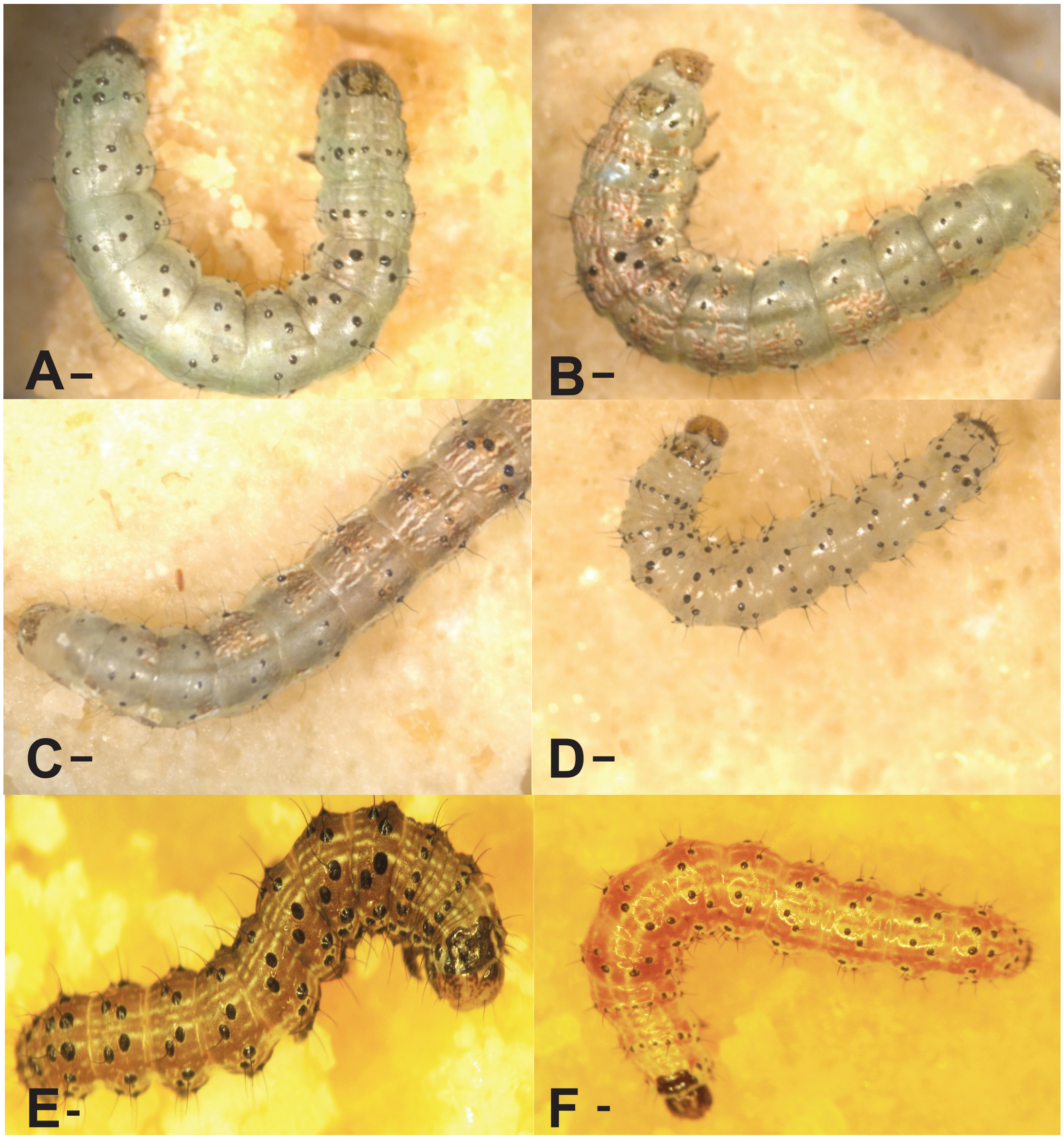

Supplementary Figure 4. DNA sequences of CRISPR-Cas9 induced mutations at the *white* locus of G0 moths. DNA sequences showing the deletion and insertions. Specific PCR products obtained using *Ha-w* gene specific primers (Supplementary Table 1) flanking the target site are shown. DNA from three different independent G0 mosaic individuals was used. Each individual showed compound insertions and deletions. Numbers to the left of the sequences indicate the loss (-) or gain (+) of bases for each allele. Darker background color indicates worse sequence quality.

Family#1 male 47e

```
wild type  AGCCGCCTACCCAGGCGAACTGCTCGCCATCATGGGATCCTCTGGTGCCGGGA
(-3)       AGCCGCCTACCCAGGCGAACTGCTCGCCAT:::GGGATCCTCTGGCGCCGGGA
(7,+2)     AGCCGCCTACCCAGGCGAACTGCT:::C:T:::GGGATCCTCTGGCGCCGGGA
(-12)      AGCCGCCTACCCAGGCG:A:::::::::::CATGGGATCCTCTGGCGCCGGGA
(-18)      AGCCGCCTACCC::::::::::::::::::CATGGGATCCTCTGGCGCCGGGA
(-10)      AGCCGCCTACCCAGGCGAAC::::::::::CATGGGATCCTCTGGCGCCGGGA
(-17)      AGCCGCCTACCCA:::::::::::::::::CATGGGATCCTCTGGCGCCGGGA
(0)        AGCCGCCTACCCAGGCGAACTGCTCGCCATCATGGGATCCTCTGGCGCCGGGA
(0)        AGCCGCCTACCCAGGCGAACTGCTCGCCATCATGGGATCCTCTGGCGCCGGGA
Target                  GGCGAACTGCTCGCCATCAT
```

Family#18 male 11e

```
wild type  AGCCGCCTACCCAGGCGAAC:TGCTCGCCAT::CATGGGATCCTCCGGTGCCGGGA
(-3)       AGCCGCCTACCCAGGCGAAC:TGCTCGCCAT::CATGGGATCCTCTGGCGCCGGGA
(+2)       AGCCGCCTACCCAGGCGAAC:TGCTCGCCATGGCATGGGATCCTCTGGCGCCGGGA
(->27)     AGCCGCCTACCCAGGCGAAC:TGCTCGCC:::::::::::::::::::::::::::
(+1,-14)   AGCCGCCTACCCAGGCGAACATGGGATCCT::::::::::::::CTGGCGCCGGGA
(-4)       AGCCGCCTACCCAGGCGAAC:TGCTCGCCA::::ATGGGATCCTCTGGCGCCGGGA
(-5)       AGCCGCCTACCCAGGCGAAC:TGCTCGCCA:::::TGGGATCCTCTGGCGCCGGGA
(-4)       AGCCGCCTACCCAGGCGAAC:TGCTCCCCA::::ATGGGATCCTCTGGCGCCGGGA
(-5)       AGCCGCCTACCCAGGCGAAC:TGCTCGCCA:::::TGGGATCCTCTGGCGCCGGGA
(-12,+2)   AGCCGCCTACCCAGGCGAAC:::::::TC:::::ATGGGATCCTCTGGCGCCGGGA
(-4)       AGCCGCCTACCCAGGCGAAC:GGCTCGCCA:::::TGGGATCCTCTGGCGCCGGGA
(-14)      AGCCGCCTACCCAGGCGAAC::::::::::::::ATGGGATCCTCTGGCGCCGGGA
(-4)       AGCCGCCTACCCAGGCGAAC:TGCTCGCCA::::ATGGGATCCTCTGGCGCCGGGA
Target                  GGCGAAC:TGCTCGCCATCAT
```

Family#17 female 10e

```
wild type  AGCCGCCTACCCAGGCGAACTGCTCGCCATCAT:::::::GGGATCCTCCGGTGCCGGGA
(0)        AGCCGCCTACCCAGGCGAACTGCTCGCCATCAT:::::::GGGATCCTCTGGCGCCGGGA
(0)        AGCCGCCTACCCAGGCGAACTGCTCGCCATCAT:::::::GGGATCCTCTGGCGCCGGGA
(0)        AGCCGCCTACCCAGGCGAACTGCTCGCCATCAT:::::::GGGATCCTCTGGAGCCGGGA
(-3)       AGCCGCCTACCCAGGCGAACTGCTCG:C::CAT:::::::GGGATCCTCTGGCGCCGGGA
(-3)       AGCCGCCTACCCAGGCGAACTGCTCG:C::CAT:::::::GGGATCCTCTGGCGCCGGGA
(-3)       AGCCGCCTACCCAGGCGAACTGCTCG:C::CAT:::::::GGGATCCTCTGGCGCCGGGA
(0)        AGCCGCCTACCCAGGCGAACTGCTCGCCATCAT:::::::GGGATCCTCTGGCGCCGGGA
(0)        AGCCGCCTACCCAGGCGAACTGCTCGCCATCAT:::::::GGGATCCTCGGGCGCCGGGA
(0)        AGCCGCCTACCCAGGCGAACTGCTCGCCATCAT:::::::GGGATCCTCTGGCGCCGGGA
(-10,+5)   AGCCGCCTACCCAGGCGAA::::::::::CCAGA:::::::GGATCCTCTGGCGCCGGGA
(0)        AGCCGCCTACCCAGGCGAACTGCTCGCCATCAT:::::::GGGATCCTCTGGCGCCGGGA
(+7)       AGCCGCCTACCCAGGCGAACTGCTCGCCATCATCGTCCATGGGATCCTCGGGCGCCGGGA
Target                  GGCGAACTGCTCGCCATCAT
```

Supplementary Figure 5. *H. armigera* genome assessed for potential off target mutations induced by crRNA against the *Ha-w* locus. (**A**) Potential off-target sites identified in *H. armigera* (Supplementary Table 3) for crRNA against *Ha-w*, contig 7769 (coding for 3-oxoacyl mitochondrial-like synthase) was examined for off-target mutations. Family#1 male 47e was used as target (Supplementary Table 2). (**B**) Similarly contig 1484 (coding for transient-receptor like protein) was examined for off-target mutations. PAM sequence in target site is underlined as red. Family#1 male 47e was used as target (Supplementary Table 2). Darker background color indicates worse sequence quality. Bases are indicated with different colors.

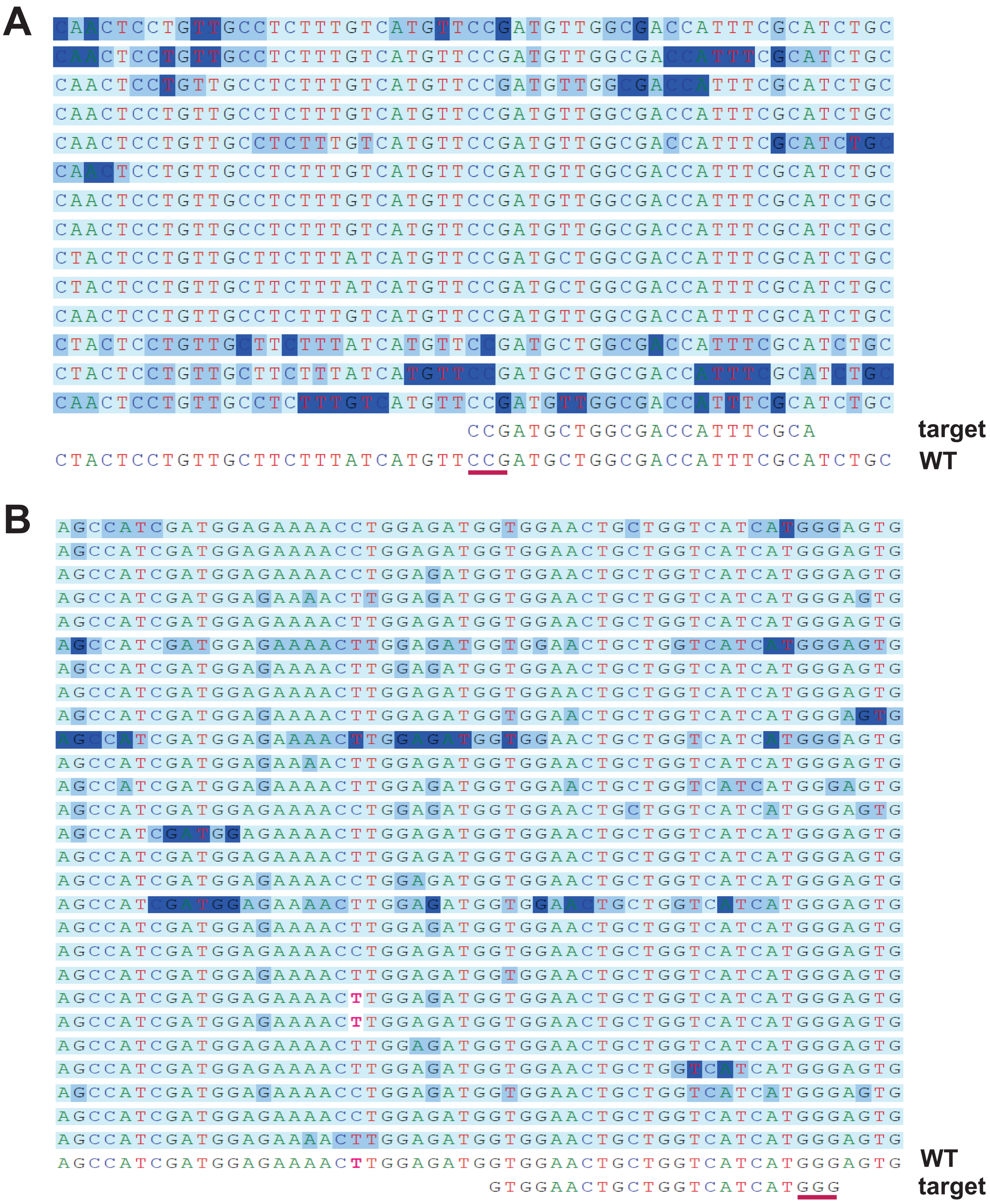
A
CCGATGCTGGCGACCATTTCGCA target
CTACTCCTGTTGCTTCTTTATCATGTTCCGATGCTGGCGACCATTTCGCATCTGC WT
B
AGCCATCGATGGAGAAAACTTGGAGATGGTGGAACTGCTGGTCATCATGGGAGTG WT
GTGGAACTGCTGGTCATCATGGG target

Supplementary Figure 6. G3 individual phenotypes of heterozygotes for the $W^{l\text{-}119}$ mutation. (**A**) Larvae have two head capsules, when molting from 3rd instar to 4th instar. These larvae were not able to shed the old head capsule. (**B**) Larva with unusual head size. (**C**) Control larva with typical head size. (**D**) Cuticle with faint brown color and reduced uric acid accumulation. (**E**) Wet larval phenotype. (**F**) Specific PCR products of the targeted region showing 3-nucleotide deletion. DNA from a single larva with the double-head phenotype) was PCR amplified and sequenced. The sequences indicate a heterozygote allelic mutation of the larva. The deleted single amino acid is marked in red. (**G**) Using a Surveyor nuclease assay, mutations were detected in the PCR products cleaved by Surveyor nuclease as indicated in the figure. DNA was taken from adults used in the cross, and their progeny had phenotype. M (50 bp DNA ladder), 1 (Ha-$W^{l\text{-}119}$ male), 2 (Ha-$W^{l\text{-}119}$ female), 3 (Control), 4 (Control), 5 (Ha-$W^{l\text{-}119}$ offspring). [a (PCR product size 257 bp), b & c digested PCR product ~160bp and 98 bp respectively]. (H) Heterozygous surviving adult and (**I**) wild type moth. Darker background color indicates worse sequence quality.

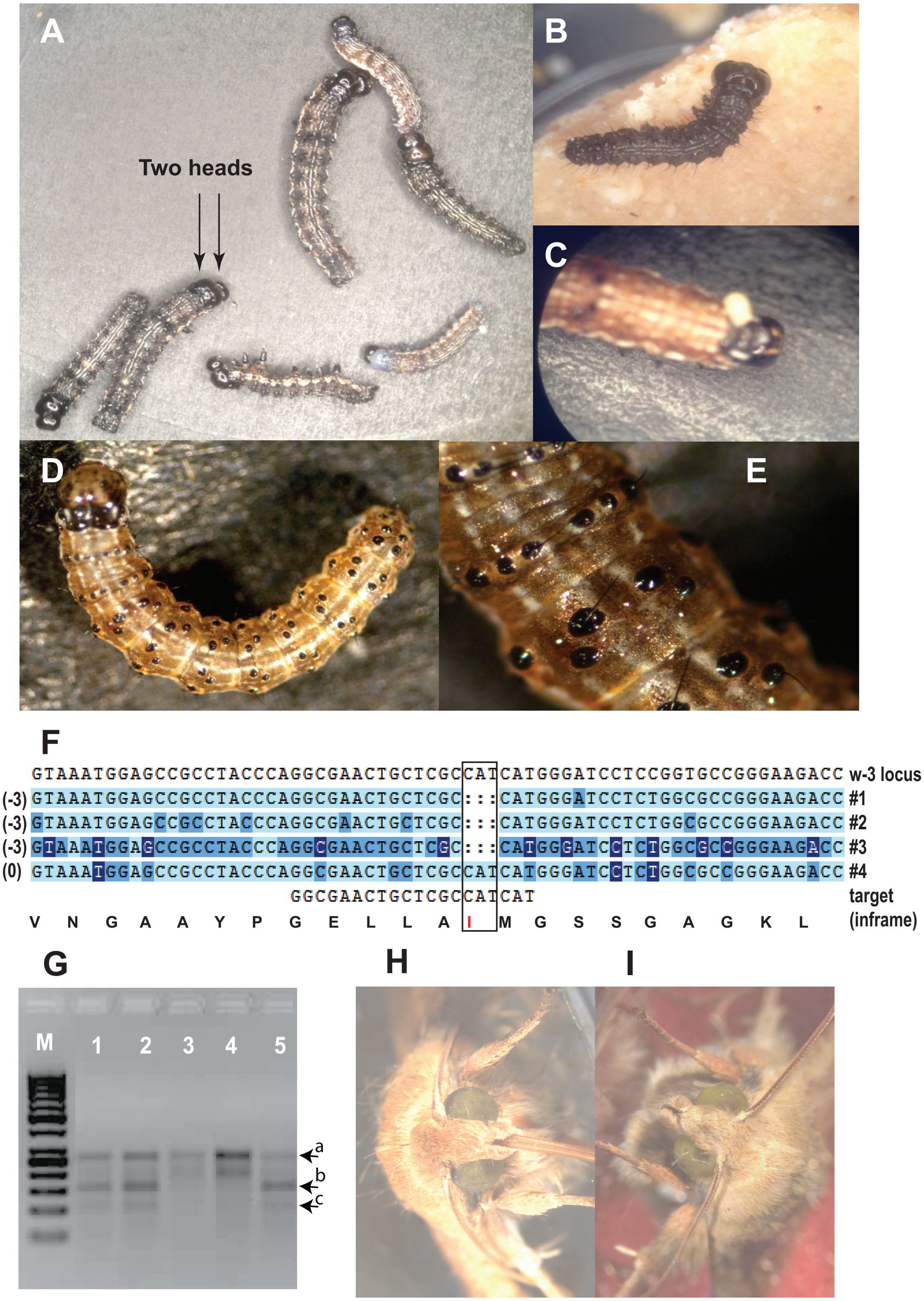

A
Two heads
B
C
D
E
F
GTAAATGGAGCCGCCTACCCAGGCGAACTGCTCGCCATCATGGGATCCTCCGGTGCCGGGAAGACC w-3 locus
(-3) GTAAATGGAGCCGCCTACCCAGGCGAACTGCTCGC:::CATGGGATCCTCTGGCGCCGGGAAGACC #1
(-3) GTAAATGGAGCCGCCTACCCAGGCGAACTGCTCGC:::CATGGGATCCTCTGGCGCCGGGAAGACC #2
(-3) GTAAATGGAGCCGCCTACCCAGGCGAACTGCTCGC:::CATGGGATCCTCTGGCGCCGGGAAGACC #3
(0) GTAAATGGAGCCGCCTACCCAGGCGAACTGCTCGCCATCATGGGATCCTCTGGCGCCGGGAAGACC #4
GGCGAACTGCTCGCCATCAT target
V N G A A Y P G E L L A I M G S S G A G K L (inframe)
G
M 1 2 3 4 5
a
b
c
H
I

Supplementary Figure 7. Protein sequence alignment of the White protein in insects. Conserved residues involved in ATP binding and hydrolysis. 1) *Anopheles gambiae* 2) *Apis mellifera* 3) *Bombyx mori* 4) *Drosophila melanogaster* 5) *Tribolium castaneum* 6) *Helicoverpa armigera* 7) *Mutant Ha-W*^{119}. The arrow indicates the position of the deleted residue.

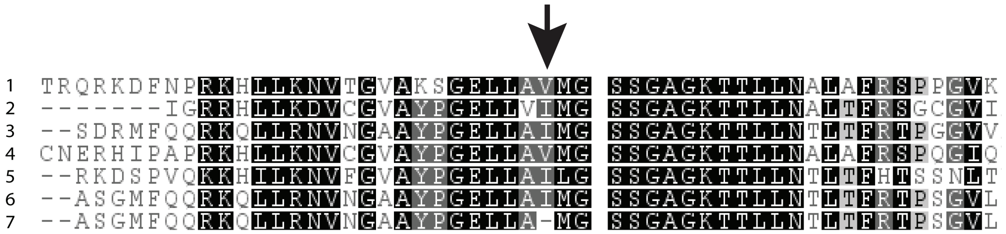

Supplementary Figure 8. Entire three-dimensional homology models of wild-type White and mutant W$^{l\text{-}119}$ proteins. (**A**) The White protein ABC transporter with white ribbon color is modeled on FbpC ABC transporter (DOI:10.2210/pdb3fvq/pdb) with magenta ribbon color using the MOE program. The third N-terminal amino acid (Alanine) of FbpC ABC transporter is shown in space filling atom style while the last two C-terminal amino acids (asparagine and isoleucine) of White protein are shown in stick atom style.(**B**) Three-dimensional model of W$^{l\text{-}119}$ protein. The W$^{l\text{-}119}$ protein ABC transporter with blue ribbon color is modeled on FbpC ABC transporter (DOI:10.2210/pdb3fvq/pdb) with golden ribbon color using the MOE program. The third N-terminal amino acid (Alanine) of FbpC ABC transporter is shown in space filling atom style while the last two C-terminal amino acids (leucine and threonine) of Ha-W$^{l\text{-}119}$ are shown in stick atom style. Amino acids Alanine 118 and Methionine 119 are missing the Isoleucine amino acid between them, which resulted in the deformed beta sheet in the Ha-W$^{l\text{-}119}$ protein.

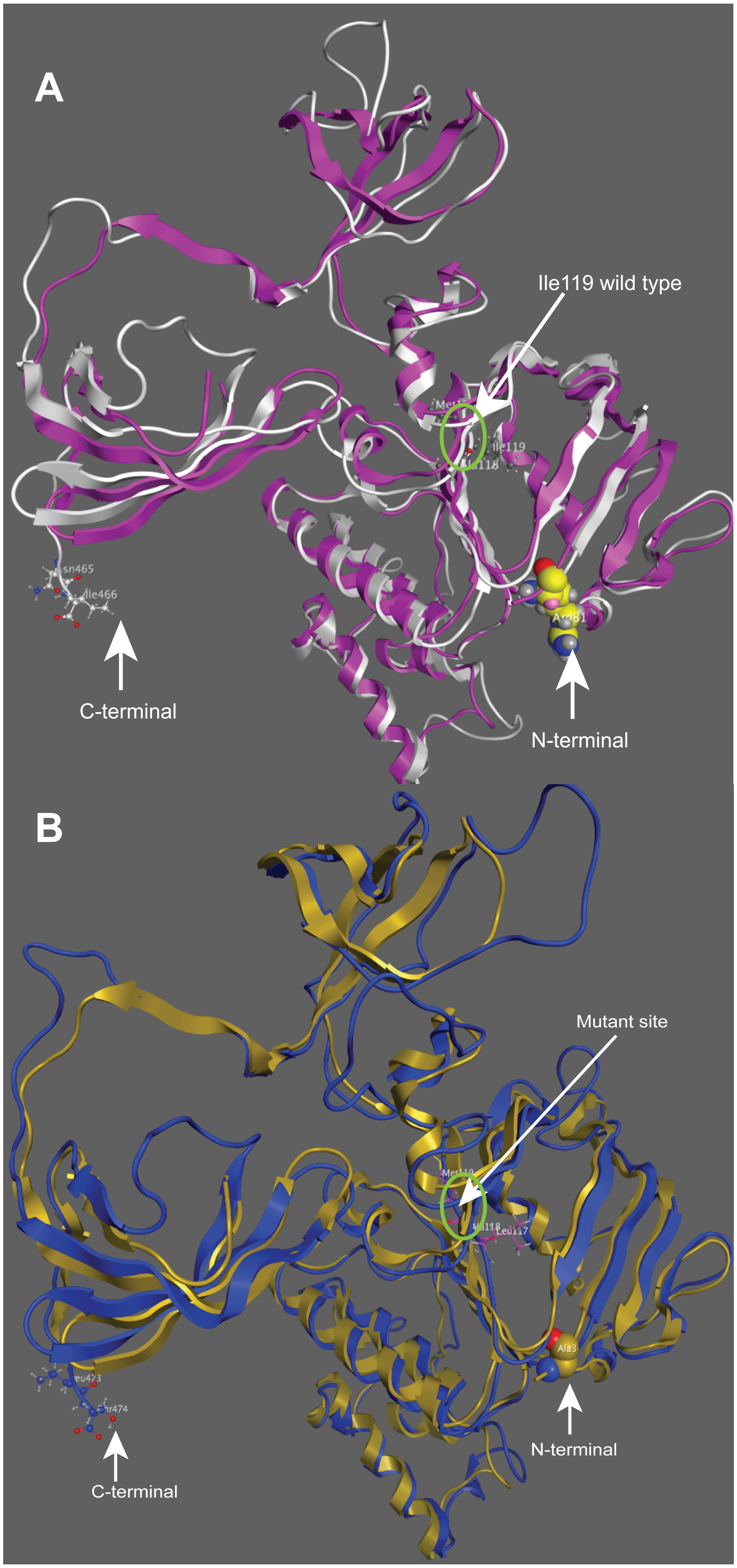
A
Ile119 wild type
C-terminal
N-terminal
B
Mutant site
C-terminal
N-terminal

Supplementary Figure 9. Sequence analysis of homozygous G2 adult yellow-eyed *scarlet* mutants. Sequences of six individuals indicating mutations at the *Ha-st* locus in G2 progeny. The mutation is due to the addition of two bases, which resulted in the in-frame shift and introduced a premature stop codon. Darker background color indicates worse sequence quality.

Adult1	CATCGGAGGCCTGGATGGAAGGAAGACCTTGTCGAGGGTGGCGAGAGAAAGAGACTG
Adult2	CATCGGAGGCCTGGATGGAAGGAAGACCTTGTCGAGGGTGGCGAGAGAAAGAGACTG
Adult3	CATCGGAGGCCTGGATGGAAGGAAGACCTTGTCGCGGGTGGCGAGAGAAAGAGACTG
Adult4	CATCGGAGGCCTGGATGGAAGGAAGACCTTGTCGAGGGTGGCGAGAGAAAGAGACTG
Adult5	CATCGGAGGCCTGGATGGAAGGAAGACCTTGTCGAGGGTGGCGAGAGAAAGAGACTG
Adult6	CATCGGAGGCCTGGATGGAAGGAAGACCTTGTCGAGGGTGGCGAGAGAAAGAGACTG
wild	CATCGGAGGCCTGGATGGAAGGAAGACCTTGTC:A:GG**T**GG**C**GAGAGAAAGAGACTG
Target	GGAAGGAAGACCTTGTC:A:GG

Supplementary Figure 10. Sequence analysis of *brown* gene (*Ha-bw*) at G0 adults and black eye mutant (*Ha-ok*) at G1 homozygous adults. (**A**) Sequence of somatic cell mutations from G0 moths. (**B**) Sequence of wild type, target site and mutated alleles around the target locus. Example of compound modified alleles from G1 homozygous male individual. Each mutated allele was named as allele 1 and 2 respectively. Number of deleted and inserted nucleotides is indicated in brackets on the right side of each allele. (**C**) Example of compound modified alleles from G1 homozygous female individual. Each mutated allele was named as allele 3 and 4 respectively. Darker background color indicates worse sequence quality.

A

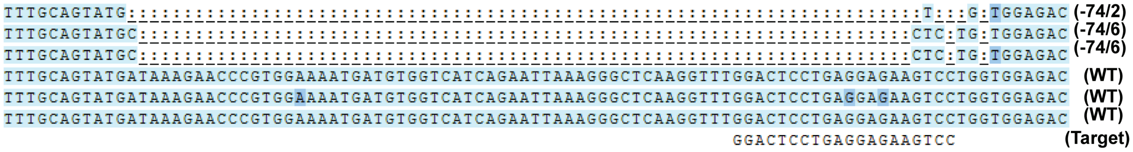

B

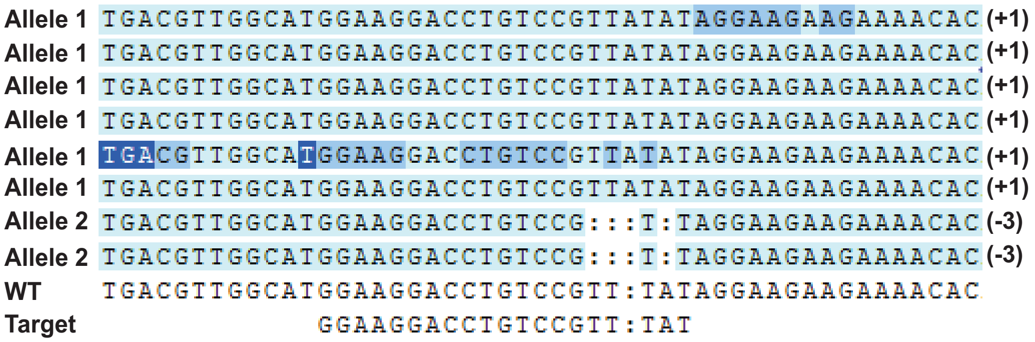

C

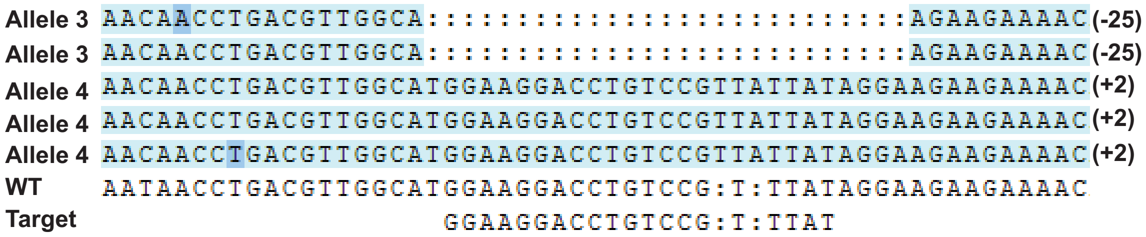

Supplementary Figure. 11. Ekapterin MS spectra from eye of the wild type individual. (**a**) MS^2 and MS^3 spectra of ekapterin (m/z 270 for $[M+H]^+$) in positive ionization mode (**b**) MS^2 and MS^3 spectra of ekapterin (m/z 268 for $[M-H]^-$) in negative ionization mode.

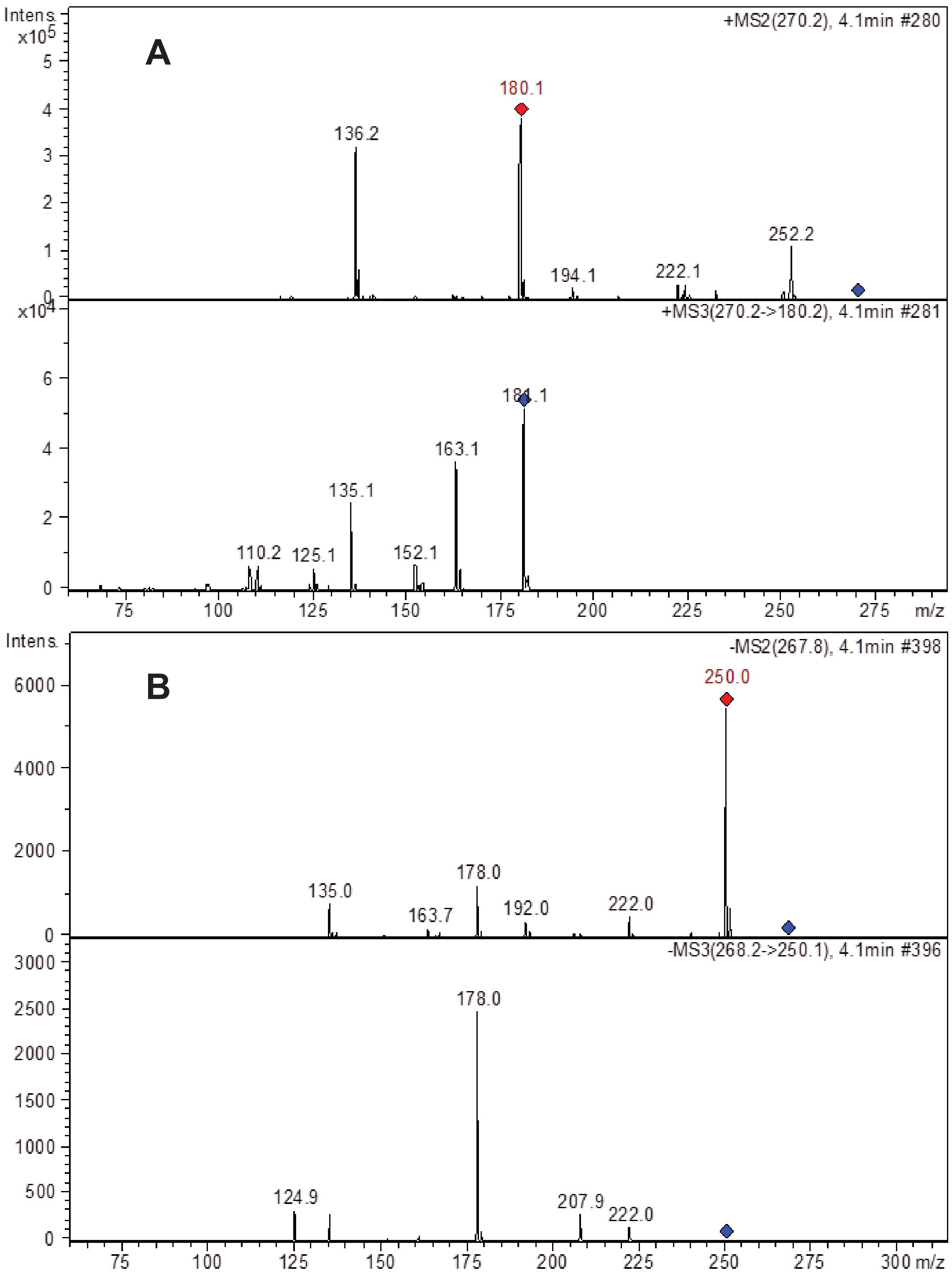

Supplementary Figure 12. Molecular phylogeny of ABC eye color transporter orthologs. Phylogeny of ABC eye color transporter orthologues showing relationships among different orthologous groups. *white* orthologues are in the white box, *scarlet* orthologues are in the red box, *brown* orthologues are in brown box and *ok* orthologues are in the tan colored box. Bootstrap consensus values are given for each node.

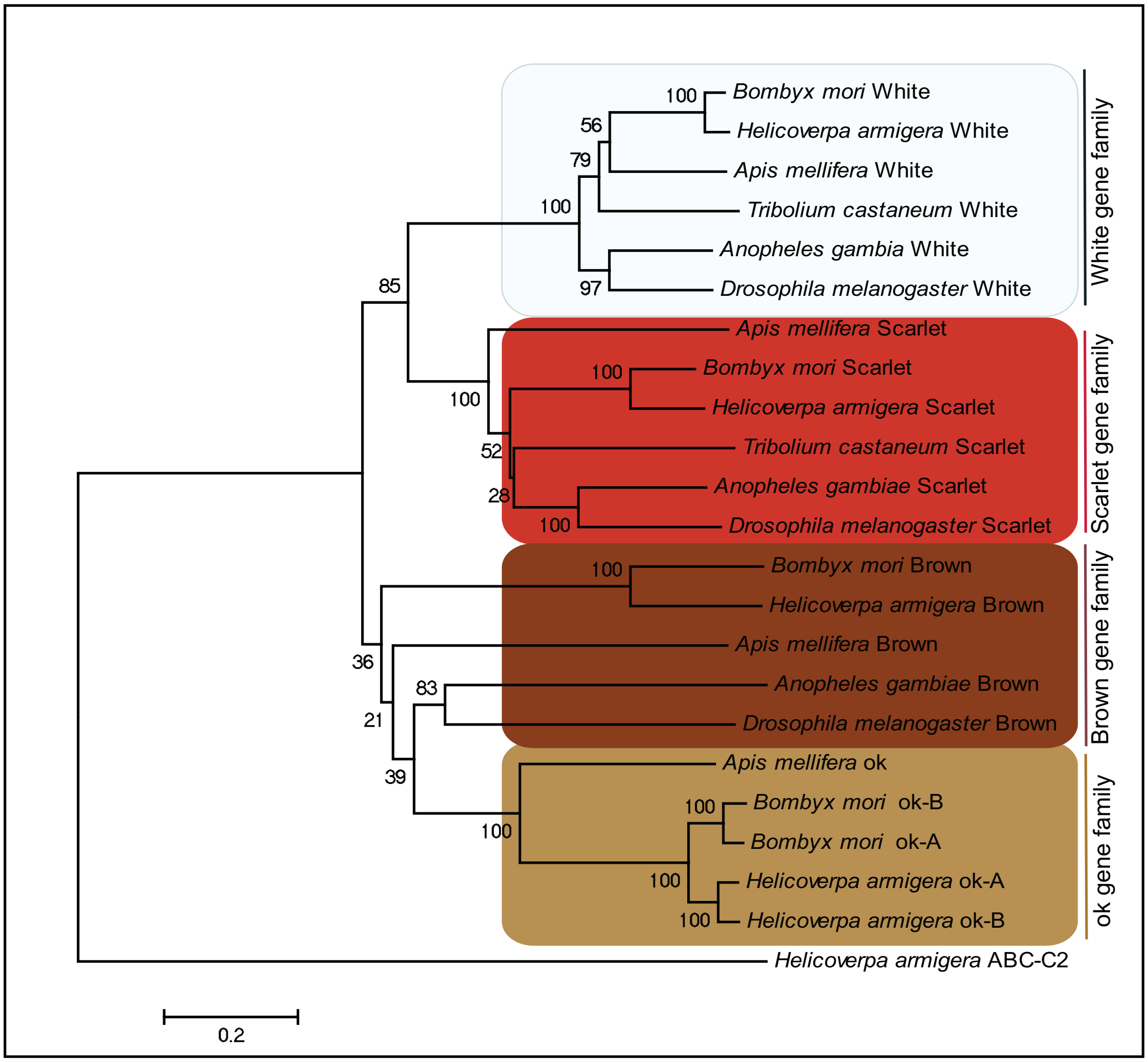

Supplementary Table 1 List of target sites and PCR primers used for mutation characterization.

Name	Target sequences	Fwd primer	Rev primer
Ha-w	GGCGAACTGCTCGCCATCAT	TGGAACTGACTTCCTCCTTCTC	GCTGCTGAACATACGCAGAC
Ha-st(1)	GGAAGGAAGACCTTGTCAGG	AGCTCGTAAGAGACGGGTGA	GGCAAATGCCAGTCTCTTTC
Ha-st(2)	GGAAGGAAGACCTTGTCGGG	AGCTCGTAAGAGACGGGTGA	GGCAAATGCCAGTCTCTTTC
Ha-bw	GGACTCCTGAGGAGAAGTCC	ACCGTGTTGTTTGCAGTATGA	TACTCTTAGTAGGTTTCCGTCTCCA
Ha-ok(1)	GGAAGGACCTGTCCGTTTAT	TGCAAATGACGTAACACAGACC	CTCCATGTAASACCCTAATTTCTTC
Ha-ok(2)	GGAAAGACCTGTCCGTTTAT	TGCAAATGACGTAACACAGACC	CTCCATGTAASACCCTAATTTCTTC

Supplementary Table 2. Phenotypes induced by microinjection of Cas9/sgRNA against the *Ha-w* locus and its effect on embryos in germ-line inheritance

Family#	male	female	Egg phenotype	Larvae separated
1	47e	43e	No egg	No larva
2	30e	49e	White egg	No larva
3	26e	54		
4	32	64	White egg	No larva
5	27e	40		
6	42	61	White egg	No larva
7	53	78e	White egg	No larva
8	60	36		
9	55e	33		
10	46	41		
11	49	51	White egg	No larva
12	57	52		
13	62	68e		
14	21e	7e		
15	6e	4		
16	70	23		
17	76e	10e		
18	11e	77e		
19	3e	39	White egg	No larva
20	50e	16e	White egg	No larva
21	18e	5e		

* “e” next to the number assigned to each adult means the individuals showed chimeric eye phenotypes. From these crosses 28% families laid eggs, which remained white in color for a week and did not hatch. The rest of the families laid eggs with wild type phenotypes and hatched normally.

Supplementary Table 3. Off-target sites and PCR primers used for mutation detection

Name	fmcontig 1484: exon2, transient receptor-like protein, nonessential	fmcontig 7769: exon 1 3-oxoacyl-[acyl-carrier-protein] synthase, mitochondrial-like (LOC101747135), nonessential
Target site	GTGGAACTGCTGGTCATCAT	TGCGAAATGGTCGCCAGCAT
Fwd primer	CACCGCAAGAAACACATA	TGGATTGATCCCTTCAGAGAA
Rev primer	GTAAGGCTCTCCCTCCTT	CAGCACCTTCACCCATGAC

Supplementary Table 4. Potential off-target sites for cRNA against *Ha-w* locus in *H. armigera* genome. The red letters indicate the variation of off-target sites from on-target site (*Ha-w* contig61482). Extreme right in target site the first three letters represents the PAM sequence.

Target sites	Contigs	start	end	strand	Hits
GGCGAACTGCTCGCCATCATGGG	Contig61482	2495	2517	+	Exon
TTAGAAAGGCTCGCCATCATTGG	Contig84514	12616	12638	-	Intron
GTGGAACTGCTGGTCATCATGGG	Contig 2009	6793	6815	+	Intron
GTGGAACTGCTGGTCATCATGGG	Contig1484	4929	4951	-	Exon
ATCCAATTGCTGGCCATCATCGG	Contig58521	5233	5255	+	Intron
ATCCAATTGCTGGCCATCATCGG	Contig37465	6529	6551	-	Intron
ACTGACCTTCTCTCCATCATCGG	Contig2441	43	65	+	Intron
TGCGAAATGGTCGCCAGCATCGG	Contig7769	5191	5213	+	Exon
CGCGAACTGCGCGCCTTCTTCGG	Contig10700	4024	4046	+	Intron
CGCGAATGCCGCGCCTTCTTCGG	Contig7948	342	364	-	Intron

Supplementary Results

Functional analysis of the ABCs of eye color in *Helicoverpa armigera* with CRISPR/Cas9-induced mutations.

Sher Afzal Khan, Michael Reichelt, and David G. Heckel

Phylogenetic analysis of eye color ABC transporters in insects

To identify the potential orthologue of eye ABC transports in *H. armigera*, we used the known eye ABC transporters from *B. mori* and BLASTP searched against an in-house EST database of *H. armigera*. From a tblastn search of the amino acid sequences of White, Brown, Scarlet and Ok from *B. mori*, we able to predict the same four orthologues in *H. armigera*. To compare the sequence similarity of these ABC transporters and understand their evolution, we searched the genomes of four well sequenced and extensively studied insects (*Apis, Tribolium, Anopheles* and *Drosophila*). All together these transporters fell into four distinct groups based on their relationship to the four *B. mori* transporters (Supplementary Figure S12). The amino acid sequences of these orthologues were used to construct a phylogenetic tree. Each of the six species analyzed had *white* and *scarlet* orthologues as the most tightly conserved groups. Probably these two groups have a critical role in multiple processes and a highly conserved role in eye pigment transport. The juxtapositional localization of these

white and *scarlet* orthologues is observed in *H. armigera* in a head to tail orientation.

brown is the least conserved group of this transporter family. *brown* is present in *Tribolium* but we did not use it in this comparison. *brown* group members possess long branches, suggesting a high degree of divergence, likely due to the ambiguity in use of pteridine. In *H. armigera* we also annotated a fourth eye color ABC orthologue belonging to the *brown* group. The same orthologue is present in *Bombyx* and *Apis* but is absent in *Drosophila* and *Tribolium* as shown in (Supplementary Figure S12). Furthermore, our annotation showed that, in each of these species, this fourth orthologue was located next to the *brown* gene in a juxtaposed tail to tail orientation. This group is named in *B. mori* as *ok* (*Bm-ok*)[1] . We kept the same name for the gene in *H. armigera* (*Ha-ok*) and analyzed its structure and function. Transcriptomic data showed the presence of splice variants of *ok* gene in *H. armigera*, and we named them as *Ha-ok-A* and *Ha-ok-B* (Supplementary Figure S1 C).

ABC transporters of *H. armigera*

To report the exon structure of eye ABC transporters, we used the gDNA and transcriptomic data for *H. armigera* to annotate the full length cDNA sequences. The *white* gene has 13 exons (Supplementary Figure S1 A). The *white* gene (*w*) was mutated at exon 3 and the target site is shown in Supplementary Figure S1A. The *scarlet* gene has 14 exons (Supplementary Figure S1 B) and exon 6 was used to design the gRNA. The *Ha-ok* gene has 12 exons and two splice

variants (Supplementary Figure S1 C). This was targeted at exon 2 to knock out both the splice variants. The *brown* gene that seems to be the paralogue of *ok* also has 12 exons but is quite different in exon structure. We targeted exon 2 of *brown* (Supplementary Figure S1D).

Supplementary Discussion

Functional analysis of the ABCs of eye color in *Helicoverpa armigera* with CRISPR/Cas9-induced mutations.

Sher Afzal Khan, Michael Reichelt, and David G. Heckel

In *H. armigera* as we know from this study and reported in a few other species[2], we also see an expansion of ABC transporter orthologues. Amino acid based similarity grouped these ABC transporters into four distinct groups correlating with their function. Similarly, White, Scarlet, and Brown have conserved neurological functions in *Drosophila*[3]. Brown is the most distinct protein of this group and has a less conserved role in insects. It may be that the lack of pteridine eye pigments in most of the species examined has relaxed the evolutionary constraints on members of the Brown group, permitting a greater degree of divergence. However, a conserved neurological function would call this conclusion into question. In *H. armigera* a *brown* orthologue is present, even though the function in eye pigmentation is not present. Orthologues of *brown* have likely persisted because of other roles besides pigment distribution. It was reported in *Drosophila* that brown plays a role in other tissues in addition to eye color[3]. The *ok* orthologues in *Bombyx* and *Apis* are clearly related to *brown* and

appear to form a distinct group. *Ok* in *H. armigera* serves a similar purpose to *brown* in *Drosophila* and *ok* in larval cuticle in *Bombyx*[2, 4, 5].

Supplementary References:

1. Wang L*, et al.* Mutation of a novel ABC transporter gene is responsible for the failure to incorporate uric acid in the epidermis of *ok* mutants of the silkworm, *Bombyx mori*. *Insect Biochem Molec Biol* **43**, 562-571 (2013).

2. Komoto N, Quan G-X, Sezutsu H, Tamura T. A single-base deletion in an ABC transporter gene causes white eyes, white eggs, and translucent larval skin in the silkworm *w-3(oe)* mutant. *Insect Biochem Molec Biol* **39**, 152-156 (2009).

3. Borycz J, Borycz JA, Kubow A, Lloyd V, Meinertzhagen IA. *Drosophila* ABC transporter mutants *white*, *brown* and *scarlet* have altered contents and distribution of biogenic amines in the brain. *J Exp Biol* **211**, 3454-3466 (2008).

4. Dreesen TD, Johnson DH, Henikoff S. The Brown protein of *Drosophila melanogaster* is similar to the White protein and to components of active transport complexes. *Mol Cell Biol* **8**, 5206-5215 (1988).

5. Wei W, Xin H, Roy B, Dai J, Miao Y, Gao G. Heritable Genome Editing with CRISPR/Cas9 in the Silkworm, *Bombyx mori*. *PLoS ONE* **9**, e101210 (2014).
